# Epigenetic Alterations in the Brain Associated with HIV-1 Infection and Methamphetamine Dependence

**DOI:** 10.1371/journal.pone.0102555

**Published:** 2014-07-23

**Authors:** Paula Desplats, Wilmar Dumaop, Peter Cronin, Sara Gianella, Steven Woods, Scott Letendre, David Smith, Eliezer Masliah, Igor Grant

**Affiliations:** 1 Department of Neuroscience, University of California San Diego, La Jolla, California, United States of America; 2 Department of Pathology, University of California San Diego, La Jolla, California, United States of America; 3 Department of Medicine, University of California San Diego, La Jolla, California, United States of America; 4 Department of Psychiatry, University of California San Diego, La Jolla, California, United States of America; University of Nebraska Medical Center, United States of America

## Abstract

HIV involvement of the CNS continues to be a significant problem despite successful use of combination antiretroviral therapy (cART). Drugs of abuse can act in concert with HIV proteins to damage glia and neurons, worsening the neurotoxicity caused by HIV alone. Methamphetamine (METH) is a highly addictive psychostimulant drug, abuse of which has reached epidemic proportions and is associated with high-risk sexual behavior, increased HIV transmission, and development of drug resistance. HIV infection and METH dependence can have synergistic pathological effects, with preferential involvement of frontostriatal circuits. At the molecular level, epigenetic alterations have been reported for both HIV-1 infection and drug abuse, but the neuropathological pathways triggered by their combined effects are less known. We investigated epigenetic changes in the brain associated with HIV and METH. We analyzed postmortem frontal cortex tissue from 27 HIV seropositive individuals, 13 of which had a history of METH dependence, in comparison to 14 cases who never used METH. We detected changes in the expression of DNMT1, at mRNA and protein levels, that resulted in the increase of global DNA methylation. Genome-wide profiling of DNA methylation in a subset of cases, showed differential methylation on genes related to neurodegeneration; dopamine metabolism and transport; and oxidative phosphorylation. We provide evidence for the synergy of HIV and METH dependence on the patterns of DNA methylation on the host brain, which results in a distinctive landscape for the comorbid condition. Importantly, we identified new epigenetic targets that might aid in understanding the aggravated neurodegenerative, cognitive, motor and behavioral symptoms observed in persons living with HIV and addictions.

## Introduction

Approximately 40 million people worldwide are infected with the Human Immunodeficiency Virus (HIV). HIV traffics into the central nervous system (CNS) early after infection and could be associated with a spectrum of neurobehavioral conditions ranging from minor neurocognitive disorder (MND) to HIV-associated dementia (HAD) [1], [2].

HIV involvement of the CNS continues to be a significant problem despite successful use of combination antiretroviral therapy (cART), which has decreased the incidence of HAD but has not greatly affected the prevalence of milder forms of HIV-associated neurocognitive disorders (HAND) [3]. This is probably due to several conditions, including drug resistance, cART toxicity and comorbidity factors such as aging, use of drugs of dependence and Hepatitis C virus infection [4], [5].

Multiple behavioral risk factors contribute to the transmission of HIV, including injection drug use that represents the second most risky behavior in the United States, accounting for one-third of the AIDS cases [6]. Drugs of abuse may act in concert with HIV proteins to damage glia and neurons, worsening the neurotoxicity elicited by HIV alone [7]. Methamphetamine (METH) in particular is a highly addictive psychostimulant drug, the abuse of which has reached epidemic proportions worldwide and its use is particularly high among persons with HIV infection [8]. METH use has progressively increased in frequency to become the second most abused illicit drug in the United States, and more than 35 million individuals worldwide use this drug. As a result of the association of METH use with high-risk sexual behavior, increased HIV transmission, and the development of antiretroviral drug resistance, METH plays an important role in driving the course of the HIV epidemic in the United States [9].

HIV infection and METH dependence are frequently comorbid and may have synergistic pathological effects, with preferential involvement of frontostriatal circuits [10]. HIV seropositive METH users have more cognitive abnormalities, higher plasma viral loads and more neurological damage than non-drug users [11], [12], [13], [14], and in turn have adverse affects on real-world health outcomes in HIV [15], [16]. The METH potentiation of HIV neurodegeneration is mediated by a complex variety of molecular mechanisms, including calcium deregulation, oxidative stress and inflammation [13], [14]. METH exposure was recently shown to alter DNA methylation, histone acetylation and gene expression in animal models [17], [18], implicating epigenetic mechanisms in its neurotoxicity.

Epigenetic mechanisms play an important role during the infection with retroviruses, including HIV-1, as they mediate the integration of the virus into the host genome, a crucial step in the viral life cycle [19], [20], [21]. Latent forms of HIV-1 are silenced by transcriptional shutdown with the establishment of a repressive chromatin environment at insertion sites by the recruitment of histone deacetylases [22]. In addition, viral promoters and enhancers are hypermethylated in the latent reservoirs from HIV-1- infected patients without detectable plasma viremia, while those same sites appeared hypomethylated in viremic patients [23].

Drug abuse alone is known to induce epigenetic changes in the brain which have been related to addictive behaviors [21], including alterations in histone tail modifications and DNA methylation, and the regulation of gene expression by non-coding RNAs in reward-associated brain nuclei. These changes modify neuronal plasticity and render individuals more prone to drug addiction [21], [24]. Acute exposure to amphetamines causes induction of *c-fos* and *fos-b* genes in the nucleus accumbens, which is associated with increased acetylation of histone 4 residues in their promoters [25].

Although the epigenetic mechanisms induced by either HIV-1 infection or drug abuse are somewhat defined, the molecular consequences of their co-occurrence are much less explored. The combination of HIV and METH, for example, results in greater interneuron loss and higher activation of reverse transcriptase in macrophages [26]. In rodent models, exposure to METH alters the expression of DNA methyltransferase I (Dnmt1), the enzyme involved in maintenance of DNA methylation and which is abundantly expressed in the adult brain [18]. Remarkably, the transcription of DNMT1 in human T-cells is also regulated by early expressed HIV-1 genes [27]. These examples suggest a synergistic action of HIV-1 infection and METH use that might be transduced via epigenetic mechanisms.

In the present report we provide evidence that concurrent HIV-1 infection and METH use alters the methylome of the host brain, inducing epigenetic and transcriptional changes that appear to be specific for the comorbid condition. We detected increased levels of DNMT1 on the brains of HIV-seropositive cases who used METH, which resulted in increased global DNA methylation. Analysis of the individual loci the showed differential methylation showed enrichment for neurodegenerative diseases; dopamine metabolism; and oxidative phosphorylation, pathways associated with neuronal damage and previously reported to be affected during HIV infection and METH use, and which might be associated with the aggravated neurodegenerative, cognitive, motor and behavioral symptoms observed on HIV seropositive individuals who use METH.

## Methods

### Study population

We evaluated 27 HIV seropositive cases (HIV+) from the National NeuroAIDS Tissue Consortium (NNTC) autopsy cohort (Table 1). Fourteen cases had no history of METH abuse or dependence (HIV+METH−) and 13 cases had a history of METH dependence (HIV+METH+). Subjects had standardized, comprehensive neuromedical and neurocognitive assessments within a median of 12 months before death. Neurocognitive testing methods have been described previously [28], and consisted of a battery of tests that assessed cognitive domains commonly affected in HIV disease, including learning, memory, attention, speed of information processing, abstraction, and verbal and motor skills. All autopsy brain tissue underwent standardized research neuropathology examination. Cases with a history of CNS opportunistic infections or non-HIV-related developmental, neurologic, psychiatric or metabolic conditions that might affect CNS functioning were excluded from the study.

**Table 1:** Clinical characteristics of the studied cohort.

METHAMPHETAMINE	NO	YES
History of abuse/dependence	(HIV+METH−)	(HIV+METH+)
**Number of subjects**	14	13
**Age at death, mean (SD)**	46 (6)	45 (10)
**Gender (M/F)**	13/1	11/2
**Neurocognitive status, NP rating (%)**		
Normal	1 (7)	5 (38)
NPI-O	8 (57)	2 (15)
ANI	1 (7)	0
MND	4 (29)	5 (38)
HAD	0	1 (9)
**Exposure to ARV, months (SD)**	9.2 (6)	23.3 (42)
**Log plasma HIV copy/ml, mean (SD**)	4.3 (0.9)	4.7 (1.5)
**Log CSF HIV copy/ml, mean (SD)**	2.4 (0.8)	2.6 (0.8)
**CD4+ lymphocytes/mm^3^, mean (SD)**	94 (144)	109 (237)
**METH Abuse (%)/Dependence (%)**	0 (0)/0 (0)	13 (100)/13 (100)
**Alcohol Abuse (%)/Dependence (%)**	5 (36)/2 (14)	6 (46)/8 (62)
**Cannabis Abuse (%)/Dependence (%)**	2 (14)/0 (0)	8 (62)/3 (23)
**Cocaine Abuse (%)/Dependence (%)**	1 (7)/0 (0)	4 (31)/3 (23)
**Opiate Abuse (%)/Dependence (%)**	0 (0)/1 (7)	1 (8)/1 (8)
**Sedative Abuse (%)/Dependence (%)**	0 (0)/1 (7)	2 (15)/1 (8)
**Other Drugs Abuse (%)/Dependence (%)**	0 (0)/0 (0)	2 (15)/2 (15)

NPI-O, neurocognitive impairment due to a cause other than HIV; ANI, asymptomatic neurocognitive disorder; MND, mild neurocognitive disorder; HAD, HIV-associated dementia.

### Standard Protocol Approvals, Registration and Patient Consents

We obtained postmortem brain tissue banked at the University of California, San Diego HIV Neurobehavioral Research Program (HNRP) under the California NeuroAIDS Tissue Network (CNTN) study. The parent study was reviewed and approved by the University of California, San Diego Human Research Protections Program. All subjects provided written informed consent prior to participating in the study and donating tissue.

### HIV RNA and DNA assays

DNA and RNA were extracted from frozen postmortem human frontal cortex samples using the DNeasy Blood and Tissue Mini kit and the RNeasy Lipid Tissue Mini kit (Qiagen), respectively. Quantification of viral DNA and unspliced cellular HIV RNA in the brain samples was performed by the HNRP Neurovirology Core (UCSD) as previously described [29]. Briefly, for RNA quantification, cDNA was generated using the Superscript III First-Strand Synthesis kit (Invitrogen) with specific primers targeting HIV pol. Nucleic acid was quantified by real-time PCR. Normalization was applied to the cellular input and expressed in copies per 10E06 GAPDH (for RNA) or ACTIN (for DNA) copies.

### Global DNA methylation assays

Genomic DNA was extracted from 25 mg of frozen brain tissue (frontal cortex) with DNeasy Blood & Tissue Mini kit (Qiagen). DNA methylation was measured in 200 ng of genomic DNA with the Methylamp Global DNA Methylation Quantification Ultra kit (Epigentek). Each sample was run in triplicate.

### Real-time PCR analysis of gene expression

Transcript abundance was determined by quantitative real-time PCR (qPCR) using Taqman technology (Life Technologies). Briefly, total RNA was extracted from frozen cortical samples using the RNeasy Lipid Tissue Mini kit (Qiagen) and was reversed-transcribed using RT^2^ First Strand kit (Qiagen) from 1 µg of total RNA. Real-time PCR analysis was performed using specific probes for DNMT1 (RefSeq NM_001130823; Taqman assay Hs00945875); DNMT3B (RefSeq NM_001207055; Taqman assay Hs00171876); TGFBR3 (RefSeq NM_001195683; Taqman assay Hs01114253); and NET1 (RefSeq NM_001o47160; Taqman assay Hs01087884) using Taqman Fast Advanced Master Mix (Life Technologies) on the StepOnePlus real-time PCR system (Applied Biosystems) according to the manufacturer instructions. PCR reactions were performed in duplicate. Relative quantification of gene expression was calculated using β-actin (ACTB; RefSeq NM_001101; Taqman assay Hs99999903) as an internal control and expressed as the inverse ratio to threshold cycle (1/dCt).

### DNMT1 protein analysis

DNMT1 protein levels were quantified by western blot and immnuhistochemistry as described earlier [30]. Briefly, 100 mg of frozen postmortem human frontal cortex were used to isolated the nuclear protein fractions with the Epiquik Nuclear Extraction Kit (Epigentek) as instructed by the manufacturer. After electrophoretic separation and transfer using standard conditions, the blots were probed with anti-DNMT1 (Abcam, 1∶1,000) or anti-TBP antibodies (1∶1,000, Active Motif). Immunohistochemistry was performed on paraffin-embedded postmortem human cortical sections as previously described previously using anti-DNMT1 (1∶250, Abcam).

### DNA methylation microarray

Briefly, 500 ng of genomic DNA were converted by bisulfite treatment using the EZ DNA Methylation kit (Zymo) according to the manufacturer’s instructions. Genome-wide methylation profiling was performed on 200 ng of bisulfite-treated DNA using the Illumina Infinium HumanMethylation 450 k BeadChip (Illumina) at the Case Western Reserve University Department of Genetics and Genome Sciences Genomics Core, following standardized protocols at the facility and according to the manufacturer’s instructions. Beadchips were scanned on an Illumina HiScan SQ. The methylation status of a specific CpG site was expressed as β values, calculated as the ratio of the fluorescence intensity signals of the methylated (M) and unmethylated (U) alleles, β = Max(M,0)/[Max(M,0)+Max(U,0)+100]. β values range between 0 (non-methylated) and 1 (completely methylated). The Illumina GenomeStudio Software (version 2011.1) was used to assess quality and extract the DNA methylation signals from scanned arrays. Methylation data was extracted as raw signals with no background subtraction and data was normalized to control probes present on the array. Sample methylation profiles including average β and intensity signals for methylated (M) and unmethylated (UM) probes obtained from the whole array are available as Files S1–4. Differential methylation analysis was performed using PARTEK Genomic Suite [31] after exporting normalized β values obtained from GenomeStudio methylation module. Principal component analysis (PCA) was used as quality control and to interrogate possible clustering of samples. Mixed-model multi-way analysis of variance was used to compare the individual CpG loci methylation data across different groups, similar to a previous report [32]. Method of Moments [33] was used to investigate the principal sources of variation. For the ANOVA model, METH dependence (present vs. absent), “Age” (coded as decades) and exposure to antiretroviral therapy (“ART”, On vs. Off) were used as categorical variables with fixed effects since their levels represent all conditions of interest and were influencing methylation levels. The model used was Yijkl = µ+METH_i_+Age_j_+ART_k_+ ε_ijkl_ where Yijkl represents the lth observation on the ith METH jth Age kth ART µ is the common effect for the whole experiment. εijkl represents the random error present in the lth observation on the ith METH jth Age kth ART. The errors εijkl were assumed to be normally and independently distributed with mean 0 and standard deviation δ for all measurements.

### Validation of DNA Methylation by High Resolution Melting Analysis

For the validation of microarray discovery we performed Methylation-Sensitive High Resolution Melting (MS-HRM) analysis on a group of genes showing differential methylation in the HIV seropositive (HIV+)/METH+ group, including NET1; TTL7; TGFBR3; SCN1A; UNC5D and APBA1. Primers were designed for bisulfite-converted DNA using Methyl Primer Express software (Applied Biosystems) to produce an amplicon <300 bp that overlapped the Illumina probe-set that showed differential methylation and also included neighbor CpG sites to increase the magnitude of the delta of their melting temperatures (Tm). Presence of CpGs in the primer sequence was avoided to prevent bias due to preferential amplification of the unmethylated target DNA [34]. Primer efficiency and specificity was tested by running a mock MS-HRM using 0% and 100% methylated human standard DNA (Life Technologies) and analyzing the profile of Tm peaks in the melting curve and the number and size of amplicons by gel electrophoresis. Bisulfite-converted DNA templates (40 ng) from the studied cases underwent PCR alongside with standards ranging from 0% to 100% methylation using MeltDoctor HRM Dye (Life Technologies). Melt curves of the samples were fitted to the sample curves using HRM 3.0 software (Applied Biosystems).

### Statistical analysis

Statistical analysis was performed using Student’s *t* test (unpaired; two-tailed) with a significance of p<0.05 or Mann Whitney test, as indicated. Correlation between viral RNA and global DNA methylation was calculated by Spearman’s rho. Linear regression was used to analyze the relation between loci-associated methylation levels and transcript abundance (Prism Graph Pad Software). Fold change of methylation was determined by analysis of variance multivariable analysis using Partek Genomics Suite (Partek) and computing for false discovery rate (FRD) at q<0.05 as described earlier.

## Results

### Comparison of the clinical characteristics of HIV seropositive individuals with and without concurrent METH use

In the present study we analyzed a total of 27 HIV seropositive cases (HIV+), whose clinical characteristics are presented in Table 1. The majority of cases were males, and in general the groups did not differ significantly respect to age, estimated duration of HIV-1 infection, year of death, nadir or current CD4+ T-cell count, or plasma viral load at the last antemortem visit.

Antemortem neurocognitive diagnosis identified a high rate of cognitive impairment in HIV+METH− cases (13 out of 14 cases) with mostly milder disease (mild neurocognitive disorder (MND) or asymptomatic neurocognitive impairment (ANI)). None had HIV-associated dementia (HAD). In the HIV+METH+ group, 62% individuals had cognitive impairment, with 5 cases of MND and 1 case of HAD. The relatively high rate of cognitive impairment in the non-METH cases may reflect brain injury that occurred before cART became available to these subjects, whose exposure to ARV on average was significantly shorter than the METH group (8.538±1.972; MEAN ± SEM for HIV+METH− and 39.25±14.25 for HIV+METH+; p = 0.043 per Student’s *t* test, unpaired; two tailed at p<0.05 significance).

As commonly observed for substance abuse disorders, previous history of abuse/dependence of other substances, including alcohol, Cannabis, cocaine, opiates and sedatives, was recorded for 6 out of 14 of the HIV+METH− group (43%) and for the 13 cases of the HIV+METH+ group (100%). Not all the subjects abused of all the substances (Table 1).

### METH use alters global DNA methylation in the brain of HIV seropositive individuals

**Figure 1:**
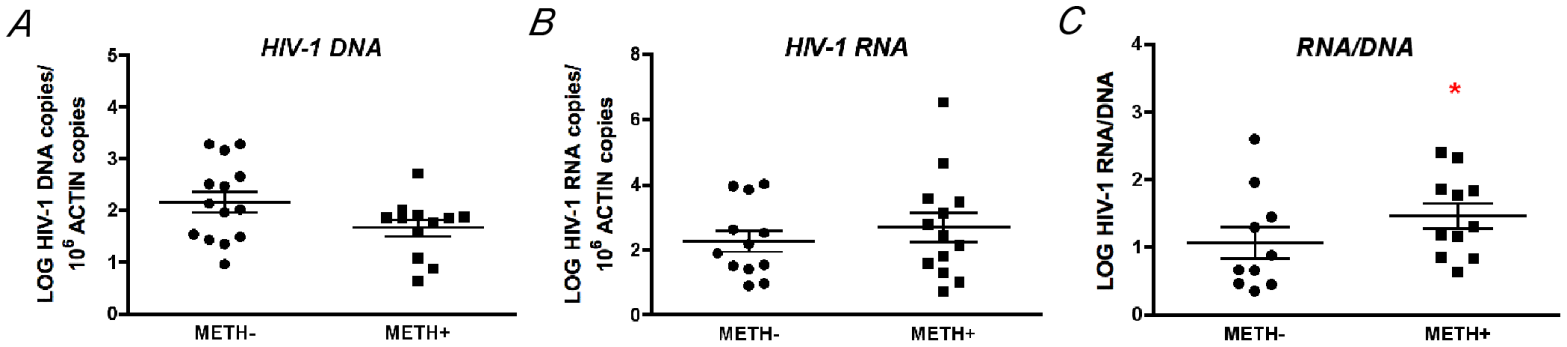
Increased HIV-1 transcription in the brains of METH-user HIV seropositive subjects. The content of HIV DNA and cellular HIV RNA was profiled in the frontal cortex area of HIV seropositive individuals with and without history of METH use, by real-time PCR and normalized to Actin or GAPDH levels respectively. ***A.*** and ***B.*** HIV-1 DNA and cellular HIV RNA did not show significant changes among groups. ***C.*** HIV-1 RNA/DNA ratio (as a marker of average HIV transcription) was significantly higher in METH user group, suggesting increases on viral transcription in the brain. *p<0.05 by Mann Whitney pairs test.

We first quantified the levels of HIV DNA and RNA content on the frontal cortex of the HIV seropositive individuals included in the study, comparing cases with and without METH use (Fig. 1). Although no significant differences were detected on HIV-1 DNA (Fig. 1A) or RNA (Fig. 1B) content in the brains of HIV+METH+ subjects in comparison to HIV+METH− individuals, a significant increase in the RNA/DNA ratio was detected in subjects who used METH (Fig. 1C), indicating higher average viral transcription associated with METH use disorders.

**Figure 2:**
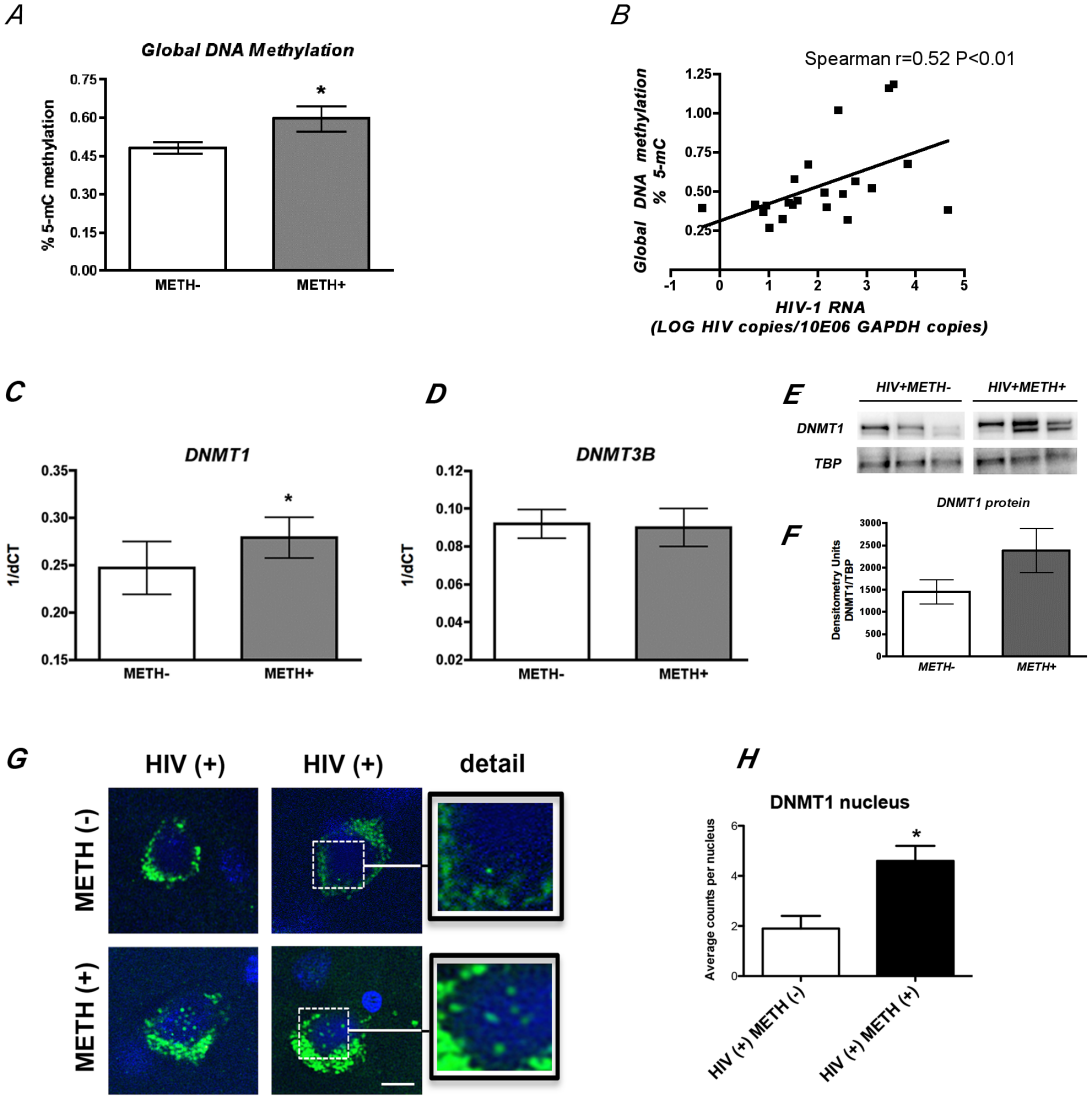
METH use results in increased global DNA methylation in the brains of HIV seropositive cases. ***A.*** Global methylation was determined by ELISA quantification of 5-mC on genomic DNA from frontal cortex samples. Significant increase in DNA methylation was observed in HIV seropositive METH users. *p<0.05 by Wilcoxon matched pairs test. ***B.*** Cellular HIV-1 RNA levels correlate with DNA methylation levels as determined by Spearman correlation between global DNA methylation and log HIV-1 RNA in the brain R = 0.527 with p = 0.01. ***C.*** Quantitative real-time PCR showed significant increase in DNMT1 transcript levels on HIV seropositive individuals who used METH. ***D.*** METH exposure did not alter mRNA levels of DNMT3B, a closely related family member reported to have redundant functions to DNMT1 in the brain. *p<0.05 by Wilcoxon matched pairs test. ***E.*** Western blot analysis of DNMT1 protein content in the nucleus showing representative HIV+METH− and HIV+METH+ cases. ***F.*** Image analysis showing integrated pixel intensity of Dnmt1 immunoreactivity. ***G.*** Immunofluorescence detection of DNMT1 on frontal cortex sections. Green fluorescent signal correspond to DNMT1 immunoreactivity and blue signal corresponds to DAPI nuclear staining. ***H.*** Image analysis showing average DNMT1 positive nuclear counts. Bar represents 10 µm. One way ANOVA was used to determine statistical significance, *p<0.05.

As HIV infection and METH use have been reported to alter epigenetic regulation, we next investigated the levels of global DNA methylation in the brain. The HIV+METH+ group had increased global methylation in the frontal cortex (Fig. 2A), which correlated with HIV RNA levels, suggesting that higher viral expression associated with METH exposure might influence DNA methylation. In agreement with previous observations on rodent models [18], the gain in methylation appears to be specifically associated with the selective increase of DNA methyl-transferase I (DNMT1) expression whose mRNA and protein levels were higher in the METH+ cases (Fig. 2C, E–H); while the groups did not differ in the levels of the close family member DNMT3B, also active in postmitotic neurons (Fig. 2D).

### Impact of Combined HIV-1 and METH on the Brain Methylome

The observed changes in global methylation might have important consequences in the host brain. We therefore explored the epigenetic changes induced by HIV disease in concert with METH dependence by profiling genome-wide DNA methylation in the frontal cortex of a subgroup of samples from our initial cohort, including 6 HIV+METH+ and 6 HIV+METH− cases. Methylome analysis was performed using the Infinium Human 450 K beadchip and GenomeStudio. For the analysis of differential methylation, we applied the Illumina Custom model after normalization to control probes present in the array and using the HIV+METH− group as the reference. We selected probes showing absolute Delta β-values>|0.2| at p<0.01 (with false discovery rate (FDR) q <0.001 to control for multiple comparisons) as differentially methylated (DM), a threshold previously suggested to improve detection of DM probes in this array platform with 99% confidence [35].

Detection levels were similar among groups, with an average of 485,381 CpG detected at p<0.01. A locus was called as detected at p<0.01 level if the mean signal intensity from multiple probes for that CpG locus was significantly higher at the level of p<0.01 than the negative control on the same chip.

**Figure 3:**
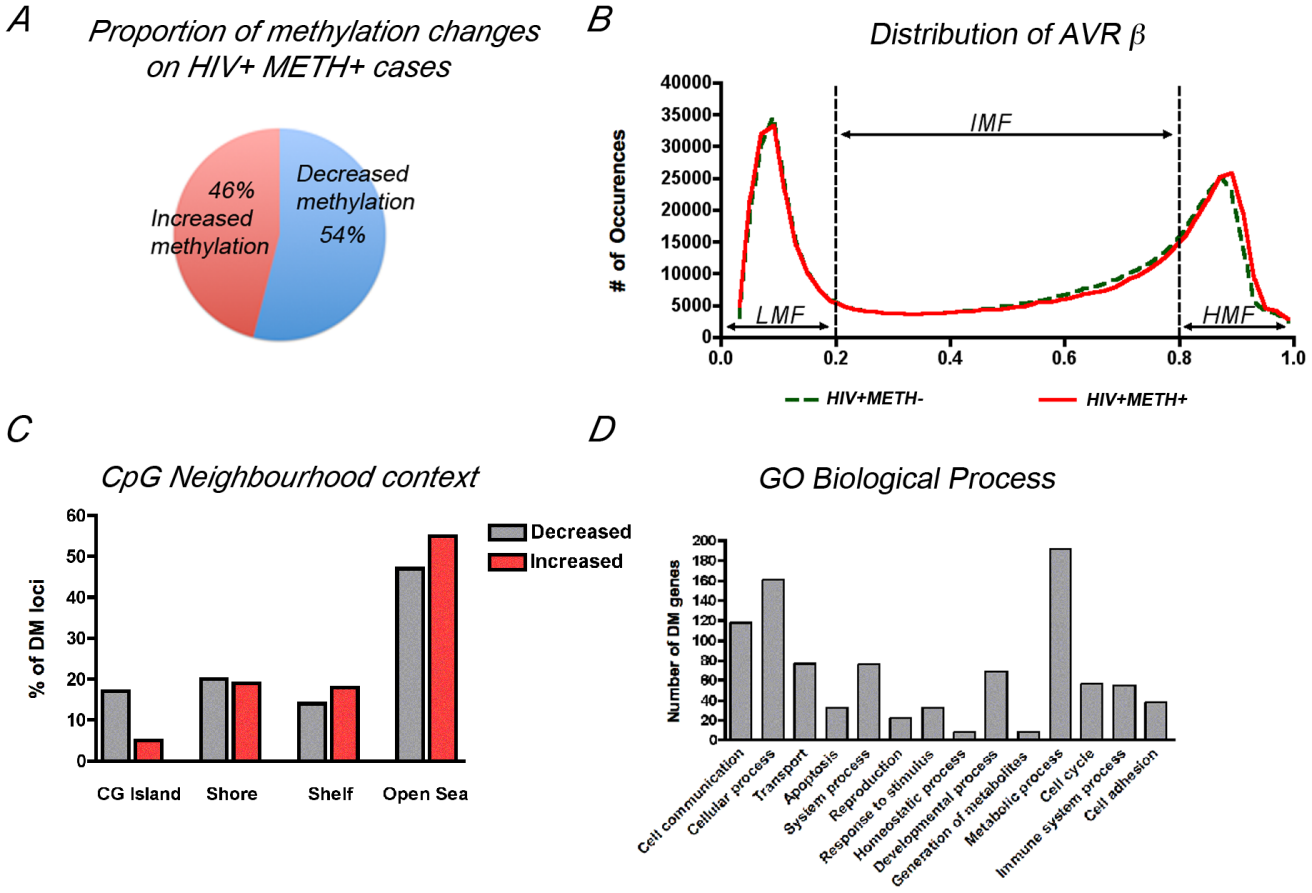
Combined HIV disease and METH dependence are associated with genome-wide DNA methylation changes in the frontal cortex. ***A*** *.* Schematic representation of the percentage of loci that showed gain or loss of methylation in the brain of HIV+ METH users as a fraction of total probes with differential methylation. ***B*** *.* Distribution of average β values across samples showing enrichment in low-methylation (LMF, β values <20%) and high-methylation fractions (HMF, β values >80%). IMF, intermediate methylated fraction (β values <20% and >80%). ***C*** *.* CpG neighborhood context analysis of loci showing differential methylation on the HIV+METH+ group. The graph represents the location of probes that showed increased methylation or decreased methylation on HIV+ METH user group (as percentage of total loci with differential methylation). CG Islands are defined as genomic regions of up to 200 bp showing enrichment of CpG dinucleotides. Shores are defined as regions up to 2 Kb from the CGi Start or End; Shelves are defined as the next 2 Kb boundaries from CGi shores. ***D*** *.* Gene Ontology analysis of annotated genes showing differential methylation clustering by Biological Process.

We first compared the methylation profiles between HIV+METH+ cases with HIV+METH− subjects. We detected 235 CpG with differential methylation, with 54% of DM loci showing decreased methylation and the remaining 46% showing increased methylation in the HIV+METH+ (Fig. 3A). Distribution of average β values across samples showed similar profiles in both groups with most CpG clustering in low-methylation (LMF, β values <20%) and high-methylation fractions (HMF, β values >80%, Fig. 3B), as previously described for another neurodegenerative disease [36]. Consistent with β-value distribution and previous reports [37], CpG neighborhood context analysis and genomic location distribution showed that loci with decreased methylation were over-represented at CG islands (CGi); while CpG sites located farther away from islands (open sea) and at the gene bodies showed increased methylation (Fig. 3 C). Finally, Gene Ontology analysis of the fraction of DM CpGs associated with annotated genes, using Panther Classification System (www.pantherdb.org) [38] and clustering by Biological Process, showed that metabolic processes, cellular processes and cell communication were the more populated clusters (Fig. 1D).

**Figure 4:**
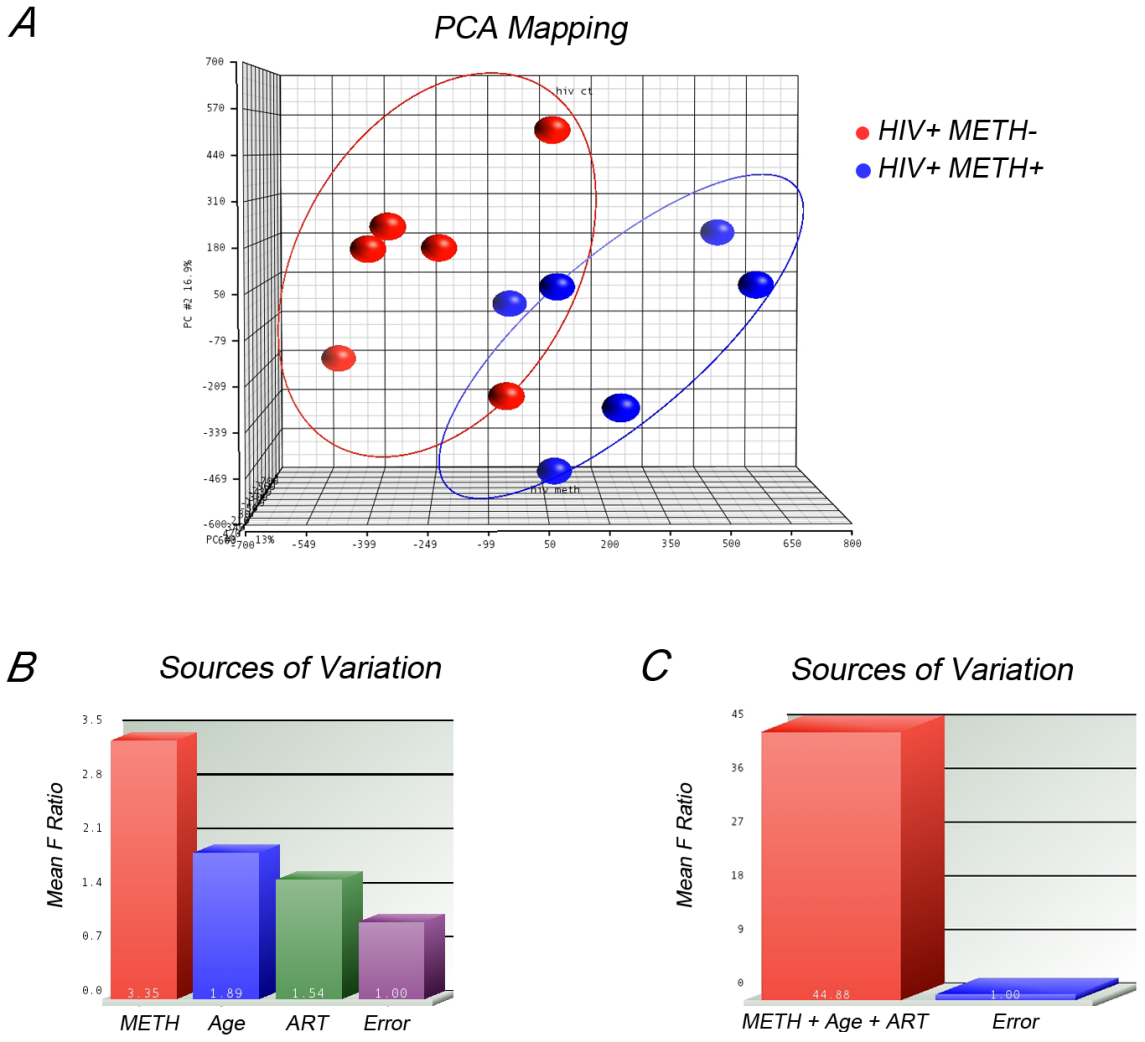
Analysis of factors that contribute to epigenetic variation. ***A.*** Principal component analysis showed clear cluster of samples only regarding METH usage status. ***B.*** Method of Moments was used to interrogate the contribution of METH use, age and antiretroviral therapy (ART) as principal sources of epigenetic variation. ***C.*** The three sources of variation were incorporated as categorical variables in a mixed-model 3-way ANOVA for the analysis of differential methylation.

In order to incorporate in the analysis other biological and clinical factors other than drug use that might modify DNA methylation, we performed a second analysis by exporting normalized β values from Genome Studio into PARTEK Genomic Suite [31]. Principal component analysis (PCA) was used as quality control and to interrogate possible clustering of samples. We tested age, exposure to antiretroviral therapy (cART) and METH dependence. METH exposure was the only characteristic that separated clusters (Fig. 4A). To further investigate the source of variation in methylation, we used multivariable ANOVA. Figures 4B–C show the significance of different sources of variation in the entire data. For the ANOVA model, METH dependence (presence or absence); age (coded as decades) and exposure to antiretroviral therapy (ART, On or Off) were used as categorical variables with fixed effect since they represent conditions of interest and influenced methylation levels.

Differential methylation analysis reporting loci with absolute fold change >1.2 at a FDR<0.05 identified a total of 446 gene-associated CGs with differential methylation in the brains of HIV+METH+ subjects, 441 corresponding to autosomal loci; with 204 presenting increased methylation (Table 2 and Table S1) and 237 showing decreased methylation (Table 3 and Table S1).

**Table 2:** Top Genes showing increased methylation (>2.0 fold) in HIV-seropositive METH users in comparison to the HIV-seropositive non-user group.

REF ACC #	Gene Name	Probeset ID	Fold change	P value	CHR
**NM_003243**	TGFBR3	cg13078798	7.84	0.00012	1
**NM_005632**	SOLH	cg26722972	5.24	0.00159	16
**NR_028415**	LOC100292680	cg21442528	4.08	0.00211	12
**NM_005539**	INPP5A	cg16645815	3.49	0.00092	10
**NM_016030**	TTC15	cg00257789	3.46	0.00055	2
**NM_003550**	MAD1L1	cg11519708	3.32	0.00006	7
**NM_005856**	RAMP3	cg23778370	3.28	0.00288	7
**NM_015307**	FAM189A1	cg21575308	3.12	0.00193	15
**NR_015451**	LOC283267	cg11008123	2.85	0.00069	11
**NM_001037631**	CTLA4	cg05092371	2.70	0.00119	2
**NM_003550**	MAD1L1	cg21598190	2.62	0.00046	7
**NM_212557**	AMTN	cg15964593	2.44	0.00251	4
**NR_002139**	HCG4	cg09565472	2.39	0.00084	6
**NM_001083308**	PYDC2	cg03883761	2.28	0.00011	3
**NM_004667**	HERC2	cg10648125	2.24	0.00079	15
**NM_017418**	“DEC1”	cg26981881	2.22	0.00073	9
**NM_018688**	BIN3	cg09094290	2.11	0.00128	8
**NM_033171**	B3GALT5	cg11479877	2.03	0.00123	21
**NR_026751**	NCRNA00171	cg11516226	2.01	0.00040	6
**NM_030883**	OR2H1	cg05111645	2.00	0.00048	6

**Table 3:** Top Genes showing decreased methylation (<−2.0 fold) in HIV-seropositive METH users in comparison to the HIV-seropositive non-user group.

REF ACC #	Gene Name	Probeset ID	Fold change	P value	CHR
**NM_052909**	PLEKHG4B	cg18816122	−14.14	0.00319	5
**NM_001052**	SSTR4	cg01471923	−8.33	0.00206	20
**NM_207189**	BRDT	cg01081438	−6.47	0.00352	1
**NM_138286**	ZNF681	cg25958450	−4.93	0.00397	19
**NM_005633**	SOS1	cg02502145	−4.76	0.00038	2
**NM_001163**	APBA1	cg14460215	−4.21	0.00229	9
**NM_024027**	COLEC11	cg10724632	−4.07	0.00015	2
**NM_002217**	ITIH3	cg05393861	−3.90	0.00085	3
**NM_021729**	VPS11	cg01385018	−3.29	0.00162	11
**NM_020728**	ESYT2	cg13211008	−3.22	0.00102	7
**NM_001165963**	SCN1A	cg00881894	−3.13	0.00048	2
**NM_001101426**	ISPD	cg11973981	−2.91	0.00333	7
**NM_001009991**	SYTL3	cg06426293	−2.91	0.00545	6
**NM_001145670**	SORBS2	cg09120722	−2.87	0.00307	4
**NM_000705**	ATP4B	cg06955954	−2.63	0.00025	13
**NM_206918**	DEGS2	cg20904336	−2.52	0.00159	14
**NM_005461**	MAFB	cg12499119	−2.37	0.00018	20
**NM_001098796**	TOX2	cg26365090	−2.31	0.00065	20
**NR_001298**	HLA-DRB6	cg25140213	−2.19	0.00170	6
**NM_020133**	AGPAT4	cg09655876	−2.16	0.00152	6
**NM_001164759**	PRKAR1B	cg10117599	−2.16	0.00002	7
**NM_080425**	GNAS	cg23484981	−2.16	0.00035	20
**NM_024686**	TTLL7	cg10977910	−2.15	0.00085	1
**NR_029411**	LOC100133091	cg17404449	−2.15	0.00046	7
**NM_001039841**	ARHGAP11B	cg18739374	−2.05	0.00113	15
**NM_001143974**	ASAH2	cg22645355	−2.01	0.00085	10

We partially verified the array findings by Methylation-Sensitive High Resolution Melting (MS-HRM) analysis, a novel form of real time PCR that uses thermal denaturation differences in bisulfite-converted DNA to create a methylation profile against known methylation standards. We tested a group of genes showing differential methylation in the HIV+METH+ cluster, including UNC5D; TGFBR3 and NET1 that were hypermethylated; and TTL7; SCN1A and APBA1 that were hypomethylated (Fig. S1). We observed coincident changes on methylation in 4 out of 6 re-tested loci.

### Epigenetic changes associated with HIV disease and METH dependence correlate with transcriptional alterations

**Figure 5:**
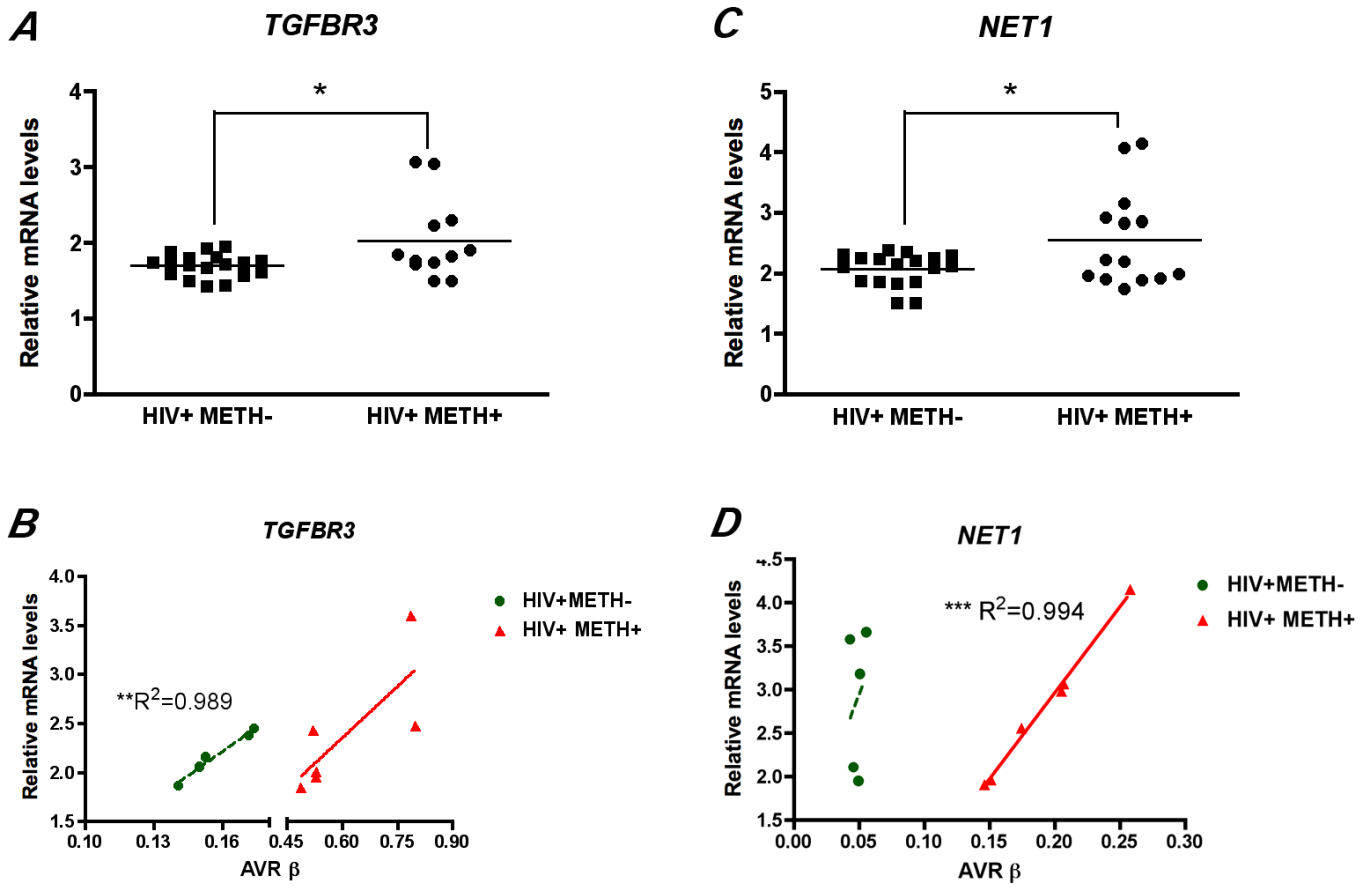
DNA methylation changes impact on the brain transcriptome. Quantitative analysis of transcript abundance by real-time PCR showed significant increases of *TGFBR3 (* ***A*** *)* and *NET1 (* ***C*** *)* on the HIV+METH+ group in comparison to HIV+METH− subjects. *p<0.05 by Student’s *t* Test. *Linear* regression analysis of gene expression and DNA methylation showed positive correlations for *TGFBR3* on HIV+METH− and on HIV+METH+ groups *(* ***B*** *)* and a significant correlation between gene expression and DNA methylation for *NET1 (* ***D*** *)*. **p<0.01 and ***p<0.001 by Linear regression analysis with a 95% confidence interval.

In order to determine the impact of these epigenetic changes on the brain transcriptome, we profiled the mRNA levels of two candidate genes whose methylation changes were validated by MS-HRM. We selected genes showing increased methylation, as we found a net gain on global DNA methylation in association with HIV disease and METH dependence (Fig. 2A). TGFBR3 had the highest change in methylation, showing a 7.8 fold increase (Table 2). We observed a significant increase in *TGFBR3* transcripts in the HIV+METH+ group (Fig. 5A). Increased DNA methylation at CG islands, which are abundant at promoter regions, is associated with repression. In contrast, methylation changes that occur at the gene body are associated with transcribed genes and directly correlated with expression [39]. The specific probe showing DM for TGFBR3 on the Illumina array mapped to a CG site not associated with the promoter or a CG island, and in agreement with the previous notion, methylation status significantly correlated with transcription in the HIV+METH− group, and showed a similar trend in the HIV+METH+ group. This suggests that methylation is involved in TGFBR3 expression on HIV+ brains, and that this activation is increased after exposure to METH (Fig. 5B). In addition we investigated NET1 transcription; a gene that also showed increased methylation (Table S1). Higher transcript levels were detected for NET1, but in this case the CG dinucleotide identified in the array analysis as hypermethylated in the HIV+METH+ group is located in a CG island, which should result in transcriptional repression (Fig. 5C). Interestingly, while linear regression analysis showed no significant association between methylation and expression in the HIV+METH− group, methylation levels strongly correlated with transcription in the HIV+METH+ group (Fig. 5D). These results suggest that the co-occurrence of HIV infection and exposure to METH might modify gene expression, not only quantitatively but also qualitatively, which might result in a more drastic deregulation on the transcriptome that the alterations induced by HIV infection alone.

### Epigenetic changes induced by METH on the brain of HIV seropositive subjects are related to neurological disease and dopaminergic alterations

To gain further insight into the biological significance of the observed epigenetic alterations, we performed data mining comparing the list of DM genes to existing datasets of genes deregulated in response to drug addiction, in particular to the abuse of amphetamine and methamphetamine [40] under the rationale that, although the observed effects on methylation can not be explained as a consequence of viral infection, which was a common factor among groups, they still might be triggered by drug exposure on itself. From a total of 117 genes previously reported altered by these stimulants, only 5 (about 4%) appeared also in our DM group, including CKB; PGM1; DPYSL2; STXBP1 and HNRNPA1. Moreover, none of the genes differentially methylated on the HIV+METH+ group were reported as altered by either alcohol, cocaine or marijuana dependence, substances that were also abused by the subjects of our cohort. This suggests that the observed changes in DNA methylation are likely due to the specific interaction of HIV-1 disease and METH dependence, rather to their individual effects or to the combined exposure to multiple substances.

We also investigated the existence of common regulatory factors that could be affecting the expression of a majority of differentially methylated genes. We performed Gene Set Enrichment analysis using the Molecular Signature Database category C3, “motif-based” to search for known and likely regulatory elements present in the promoters and 3′ UTR on either hypermethylated or hypomethylated gene groups. Interestingly, NFAT, nuclear factor of activated T cells, had the highest significance overlapping on both gene sets, with 23 genes on the hypermethylated group and 28 from the hypomethylated fraction included in the overlap at FDR q values of 6.7 E-04 and 1.9 E-05 respectively. This finding suggests that differentially methylated genes might be linked at a higher regulatory level by NFAT.

Lastly, we performed canonical pathway analysis by feeding our entire list of differentially methylated genes into Ingenuity Pathway Analysis software to interrogate if epigenetic deregulation was more likely affecting a particular biological pathway. Interestingly, Neurological Disease (illustrated in Fig. S2) and Psychological Disorders were among the highest represented groups with significant alterations (p values <0.001 in both cases), with L-DOPA Degradation (p value 3.95E-04), ERK/MAPK Signaling (p value 1.41E-03) and Dopamine-DARPP32 Feedback in cAMP Signaling (p value 1.61E-03) being the top ranked canonical pathways. These results are in agreement with neuropathology and behavioral alterations documented for HIV seropositive individuals that are exacerbated by METH use, and also with the observation of deregulation of the dopaminergic system by METH [41], highlighting DNA methylation as a molecular mechanism implicated on the neurodegeneration associated with the interaction of HIV disease and METH dependence in the brain.

## Discussion

In the present study we analyzed epigenetic changes associated with METH dependence as a comorbid presentation to HIV-1 infection. We focused on changes pertinent to DNA methylation, as this epigenetic modification is altered independently by both, viral infection and exposure to drugs. We present evidence for the existence of a unique cortical methylome induced by the interaction of HIV and METH, and which results in deregulation of many genes linked to AIDS and drug dependence neuropathologies, but which can not be fully explained by either factor alone. We proposed that changes in methylation induced early in infection by HIV potentiate the alterations in the DNA methylation machinery, particularly induction of DNMT1 expression, which is further altered after METH exposure. Our results provide a new molecular understanding of gene alterations due to the interaction of METH and HIV, standing from the host point of view.

Epigenetic mechanisms encompass DNA methylation, histone posttranscriptional modifications and non-coding RNA forms, and produce heritable changes in the genome that shape the phenotype without altering the DNA sequence. These mechanisms are intimately ligated to viral replication and to molecular changes induced by drugs of dependence.

Epigenomic regulation is implicated in integration and viral latency, two crucial steps in the HIV-1 life cycle. Integration of proviral DNA into the host genome is essential for HIV-1 replication [42], a mechanism favored by an array of epigenetic modifications including H3 and H4 acetylation and H3 and K4 methylation. In contrast, viral integration is impaired around chromatin regions harboring H3 K27 methylation and DNA methylation [43]. Moreover, epigenetic control is tightly connected with viral latency. Silencing of viral replication is achieved by the concerted recruitment of chromatin remodeling factors to the integrated LTR sites, which result in chromatin structural changes that prevent further transcription [20], [22]. We recently reported that imbalance of the epigenetic factors involved in this chromatin-remodeling complex is associated with latent HIV infection in postmortem brains [19]. In addition, integrated provirus appears to be silenced by CpG methylation around the integration sites [44]. Therefore, major epigenetic changes could be driven by HIV-1 at different stages of its life cycle, which might impact the host. Notably, a higher degree of DNA methylation at the 5′-LTR of HIV-1 has been reported for long-term “non-progressor” and “ellite-controller” patients in comparison to “progressor” cases, implicating the role of DNA methylation on viral replication and its impact on disease outcomes [45].

Epigenetic mechanisms are sensible responders to the environment and can elicit changes in cellular physiology upon exposure to varied stimuli, including use of drugs of addiction. Exposure to increasing doses of METH alters glutamatergic function on rat striatum, an effect accompanied by epigenetic changes, including increased recruitment of CoREST, HDAC2, MeCP2 and SIRT2, which downregulate GluA1 and 2 transcription [46]. In addition, a recent study aimed at understanding the epigenetic changes induced by chronic METH exposure leading to neuroadaptations at glutamatergic synapses suggested that H4 hypoacetylation may be a determinant factor of this response [47].

The neurotoxic effects of HIV-1 are in part due to its ability to enter the CNS early during infection, causing a deficiency in dopaminergic function [48]. The frontostriatal regions of the brain are highly vulnerable to neurotoxins released by infected macrophages/microglia cells [49], but this brain region is also injured by METH via increased dopamine and glutamate transmission, which further leads to neuronal damage [13]. Thus, the concomitant adverse effects of HIV-1 and METH on similar neuronal circuits exacerbate neuronal damage that each factor can produce alone. Another common pathway that is affected by both, HIV and METH is the regulation of the DNA methylation machinery itself. Early expressed HIV-1 proteins act directly on the promoter region and induce the transcription of DNMT1, without concomitant activation of DNMT3A or DNMT3B [27]. As DNMT1 is the main maintenance methylation enzyme resident in the adult brain, HIV-induced deregulation could have a substantial impact on the CNS, particularly on sustaining methylation patterns. Moreover, METH exposure was reported to alter the expression of Dnmt1 on the rat brain, by a mechanism mediated by glucocorticoid hormones, which are increased by METH. This cascade leads to differential DNA methylation and altered gene expression in the nucleus caudatus and the nucleus accumbens of exposed animals [18]. In agreement with these observations we report here specific increase of DNMT1 transcription that results on higher levels of global DNA methylation and associated with higher viral expression, suggesting that both stimulus act synergistically to reshape the epigenetic landscape in the host brain.

The experimental design of our study, in which we compared HIV seropositive individuals who only differed significantly on their use of METH, enabled us to withdraw changes induced exclusively by the virus, therefore altered methylation patterns reflect changes due to the interaction of HIV disease and METH dependence. Although the subjects included in the cohort had also a history of abuse/dependence of a variety of other substances, the fact that only 4% of differentially methylated genes identified in our study matched extensive collections of genes deregulated by drug use reinforces the uniqueness of the methylome we described here.

Noteworthy, pathway analysis including genes with altered methylation showed enrichment for neurodegenerative diseases, with particular effects on dopamine metabolism and transport, highly pertinent to the neuronal damage due to HIV and METH as described earlier. Moreover, the observation that many DM genes appear to be under co-regulated by NFAT, a factor directly linked to HIV-1 infection; suggests a complex cross talk between transcriptional and epigenetic factors that result on this signature epigenome/transcriptome. NFAT is associated with a broad spectrum of regulatory molecules, including growth factors and cytokines, and it is also important for the induction of specific genetic programs that guide differentiation and effector activity of CD4+ T helper cells and also governs the transcription of signature cytokines [50]. In addition, NFAT transcription factors can regulate HIV-1 expression by direct binding to an specific site located at the 5′ LTR [51], highlighting a fundamental role of this protein during HIV-1 infection and the relevance of the epigenetically-deregulated gene set that we report here.

In sum, we provide evidence of the role of DNA methylation as a molecular mediator of alterations induced by HIV infection and METH use, whose complexity need to be further investigated. This epigenetic analysis also unraveled novel genes that might be related to precise neurodegenerative cascades, contributing to a better understanding of the pathways affected by these highly comorbid conditions.

## Supporting Information

Figure S1 Validation of array findings by MS-HRM.
(PDF)

Figure S2 Pathway analysis showing enrichment for Neurological disease.
(PDF)

Table S1 Complete list of differentially methylated genes.
(DOCX)

File S1 Individual sample methylation profile for all interrogated probes on the array for HIV+METH− cases.
(ZIP)

File S2 Individual sample methylation profile for all interrogated probes on the array for HIV+METH− cases.
(ZIP)

File S3 Individual sample methylation profile for all interrogated probes on the array for HIV+METH+ cases.
(ZIP)

File S4 Individual sample methylation profile for all interrogated probes on the array for HIV+METH+ cases.
(ZIP)
