# Supplementary material for: Epigenetic Alterations in the Brain Associated with HIV-1 Infection and Methamphetamine Dependence
Source: PLoS One. 2014 Jul 23;9(7):e102555. doi: 10.1371/journal.pone.0102555 (PMC4108358; doi:10.1371/journal.pone.0102555)

Figure S1. Validation of array findings by Methylation-Sensitive High Resolution Melting Analysis (MS-HRM). MS-HRM is a novel form of real time PCR that uses thermal denaturation differences in bisulfite-converted DNA to create a methylation profile against known methylation standards. Primers were designed for bisulfite-converted DNA to produce an amplicon < 300 bp and overlapping the Illumina probe-set that showed differential methylation and including neighbor CpG sites to increase the magnitude of the delta Tm. ***A, C, E, G, I and K.*** Melting curves of the genes selected for validation were fitted to standard methylation curves covering 0 to 100% methylated human genomic DNA (run in the same plate) using HRM software. ***B, D, F, H, J and L.*** Comparison of individual Tm for each amplicon as determined by HRM analysis among METH abusers and non-users groups. * p<0.05; ** p<0.01 and *** p<0.001 per Student's *t* Test. Layouts of primer location in comparison to Illumina array probes coverage are depicted at the bottom of each graph: black line represent genomic region to analyze; arrows indicate the location of forward and reverse primers designed for HRM; green segment indicates Illumina probe coverage and red dots show position of profiled CpG residues.

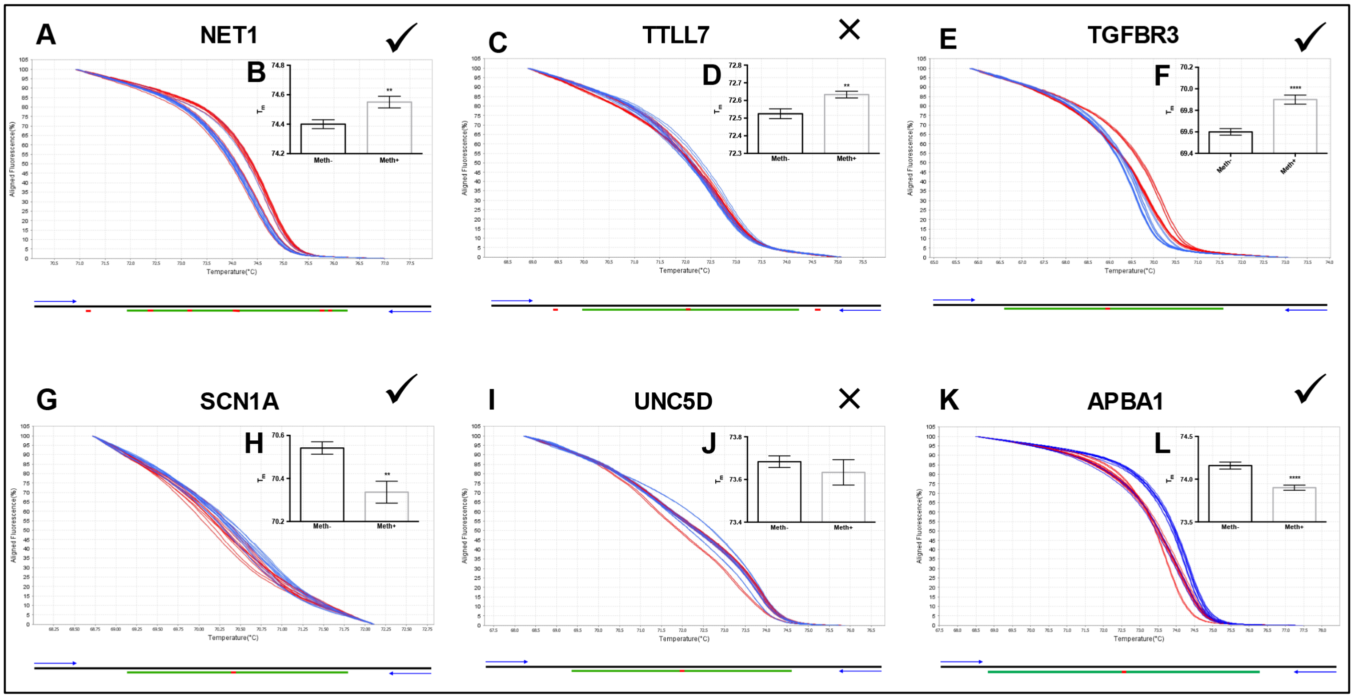

Supplement: Figure S1 — Validation of array findings by MS-HRM. (PDF) [file pone.0102555.s001.pdf]
