# Supplementary material for: Epigenetic Alterations in the Brain Associated with HIV-1 Infection and Methamphetamine Dependence
Source: PLoS One. 2014 Jul 23;9(7):e102555. doi: 10.1371/journal.pone.0102555 (PMC4108358; doi:10.1371/journal.pone.0102555)

Figure S2. Schematic depiction of Neurological Disease pathway highlighting differentially methylated genes in HIV+ METH abusers. Canonical pathway analysis was performed using Ingenuity Pathway Analysis. Genes with increased methylation are showed in red and those with decreased methylation appear in green. The association of differentially methylated genes with Neurological disease was reported with a score of 44 and p values ranging between 1.38E-04 and 1.99E-02 for all associated molecules.

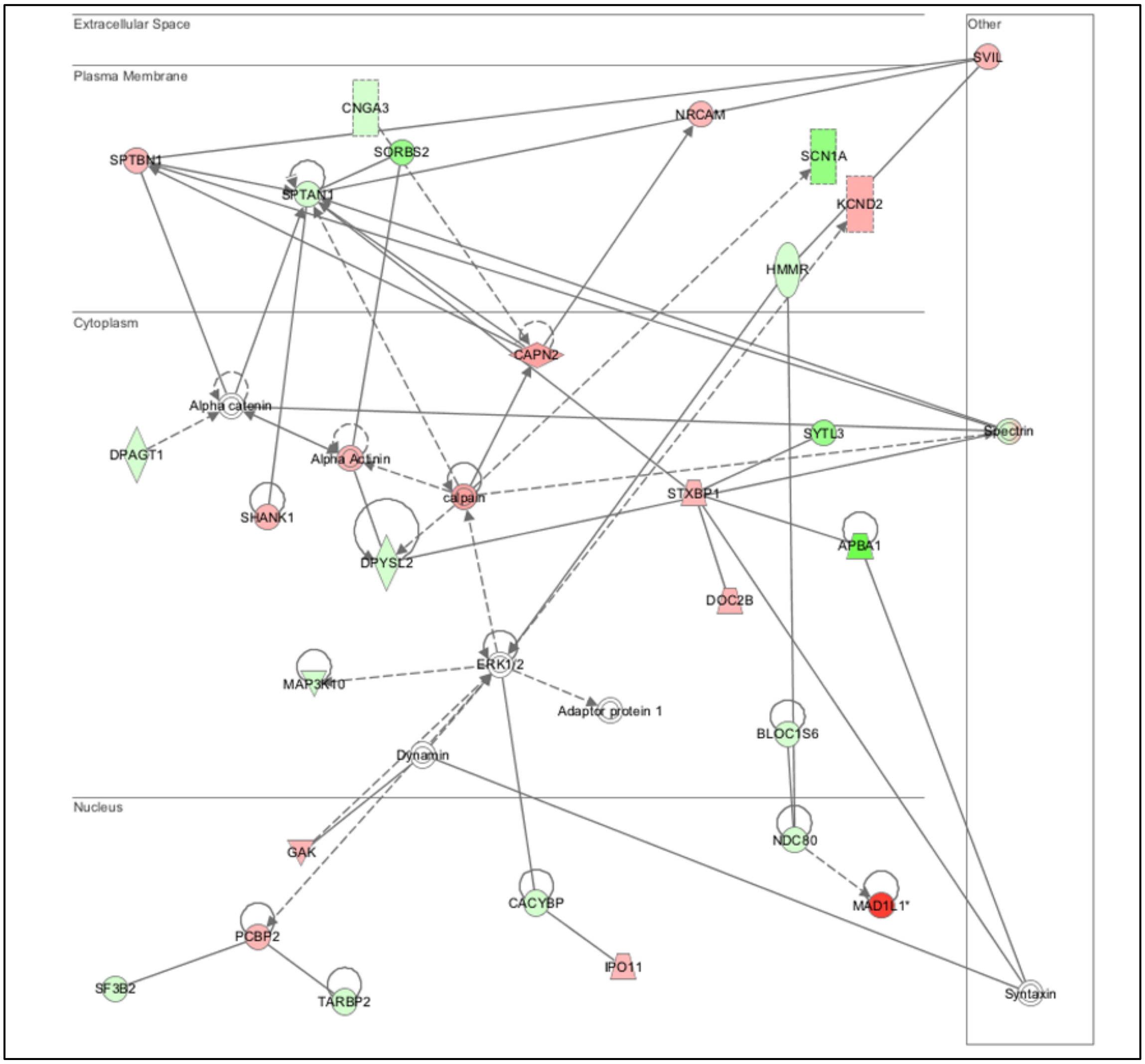

Supplement: Figure S2 — Pathway analysis showing enrichment for Neurological disease. (PDF) [file pone.0102555.s002.pdf]
