# Supplementary material for: Epigenetic Alterations in the Brain Associated with HIV-1 Infection and Methamphetamine Dependence
Source: PLoS One. 2014 Jul 23;9(7):e102555. doi: 10.1371/journal.pone.0102555 (PMC4108358; doi:10.1371/journal.pone.0102555)
Supplement: Table S1 — Complete list of differentially methylated genes. (DOCX) [file pone.0102555.s003.docx]

***Table S1. Complete list of gene-associated probes that showed significant changes on methylation on the HIV seropositive subjects who used METH, as per multi-way ANOVA analysis.***

| *CHR* | *RELATION CPG_ISLAND* | *UCSC_CPG_ISLANDS* | *UCSC_ACC#* | *REF_NAME* | *Probeset ID* | *Fold-Change* | *p-value* |
| --- | --- | --- | --- | --- | --- | --- | --- |
| *Genes with increased methylation in HIV+/METH users* | | |  |  |  |  |  |
| 1 |  |  | NM_003243 | TGFBR3 | cg13078798 | 7.83501 | 0.000121158 |
| 16 | N_Shelf | chr16:604801-605348 | NM_005632 | SOLH | cg26722972 | 5.24013 | 0.00159351 |
| 12 |  |  | NR_028415 | LOC100292680 | cg21442528 | 4.08335 | 0.00210771 |
| 10 | S_Shore | chr10:134555113-134555338 | NM_005539 | INPP5A | cg16645815 | 3.49124 | 0.000917229 |
| 2 | N_Shore | chr2:3452346-3452582 | NM_016030 | TTC15 | cg00257789 | 3.4644 | 0.000548836 |
| 7 | S_Shelf | chr7:1977286-1977912 | NM_003550 | MAD1L1 | cg11519708 | 3.3227 | 5.50E-05 |
| 7 | S_Shore | chr7:45197181-45197807 | NM_005856 | RAMP3 | cg23778370 | 3.27501 | 0.00287992 |
| 15 |  |  | NM_015307 | FAM189A1 | cg21575308 | 3.11502 | 0.00192705 |
| 11 |  |  | NR_015451 | LOC283267 | cg11008123 | 2.85288 | 0.000686201 |
| 2 |  |  | NM_001037631 | CTLA4 | cg05092371 | 2.70284 | 0.00118506 |
| 7 | N_Shore | chr7:2099668-2100358 | NM_003550 | MAD1L1 | cg21598190 | 2.61561 | 0.000459253 |
| 4 |  |  | NM_212557 | AMTN | cg15964593 | 2.43691 | 0.00250581 |
| 6 | S_Shore | chr6:29759946-29760903 | NR_002139 | HCG4 | cg09565472 | 2.386 | 0.000835375 |
| 3 |  |  | NM_001083308 | PYDC2 | cg03883761 | 2.28495 | 0.000107456 |
| 15 |  |  | NM_004667 | HERC2 | cg10648125 | 2.23763 | 0.000793204 |
| 9 |  |  | NM_017418 | "DEC1" | cg26981881 | 2.21623 | 0.000726681 |
| 8 | S_Shore | chr8:22526298-22526757 | NM_018688 | BIN3 | cg09094290 | 2.1085 | 0.00128251 |
| 21 |  |  | NM_033171 | B3GALT5 | cg11479877 | 2.03237 | 0.00122582 |
| 6 |  |  | NR_026751 | NCRNA00171 | cg11516226 | 2.01073 | 0.00039645 |
| 6 |  |  | NM_030883 | OR2H1 | cg05111645 | 1.99977 | 0.000483978 |
| 5 |  |  | NM_001159707 | BTNL8 | cg22561794 | 1.97024 | 0.00157177 |
| 17 | S_Shore | chr17:76472296-76472533 | NM_173628 | DNAH17 | cg00461299 | 1.88297 | 0.00122426 |
| 7 | N_Shelf | chr7:104885049-104885400 | NM_182691 | SRPK2 | cg00571519 | 1.8758 | 0.000867095 |
| 10 | Island | chr10:5454431-5454779 | NM_001047160 | NET1 | cg13661397 | 1.82226 | 0.000188267 |
| 15 |  |  | NM_001080490 | PLA2G4E | cg02471325 | 1.812 | 0.000343625 |
| 7 |  |  | NM_003117 | SPAM1 | cg08385371 | 1.7278 | 0.000652546 |
| 19 | Island | chr19:54040812-54041857 | NM_001079906 | ZNF331 | cg15576576 | 1.70927 | 0.000329153 |
| 11 |  |  | NM_018161 | NADSYN1 | cg09776041 | 1.69975 | 0.0018263 |
| 14 |  |  | NM_001756 | SERPINA6 | cg22827011 | 1.69672 | 0.0012421 |
| 5 | S_Shore | chr5:126409006-126409327 | NM_001164479 | FLJ44606 | cg01208126 | 1.69665 | 0.000269135 |
| 7 |  |  | NM_001100390 | IQCE | cg16686273 | 1.6774 | 0.00173405 |
| 21 | Island | chr21:43989948-43990199 | NM_018964 | SLC37A1 | cg08407901 | 1.65694 | 0.000205772 |
| 1 |  |  | NM_001114108 | TTC22 | cg13370086 | 1.64553 | 0.00051378 |
| 1 | S_Shore | chr1:108735227-108735513 | NM_213651 | SLC25A24 | cg17107246 | 1.64155 | 0.000194745 |
| 17 | S_Shore | chr17:1538415-1538851 | NM_145352 | SCARF1 | cg17028259 | 1.61538 | 0.000358674 |
| 20 | N_Shore | chr20:45279781-45280169 | NM_022829 | SLC13A3 | cg03375067 | 1.5929 | 0.000192382 |
| 1 | S_Shore | chr1:223899990-223900823 | NM_001748 | CAPN2 | cg05761882 | 1.58197 | 0.000515898 |
| 10 |  |  | NM_005308 | GRK5 | cg01516887 | 1.58125 | 0.00035485 |
| 8 |  |  | NM_080872 | UNC5D | cg22661129 | 1.58032 | 0.000382138 |
| 1 | S_Shelf | chr1:249132080-249133310 | NM_024836 | ZNF672 | cg21149260 | 1.56943 | 0.000405454 |
| 2 |  |  | NR_002712 | IL8RBP | cg19491221 | 1.56805 | 0.000197302 |
| 16 | Island | chr16:75269028-75269879 | NM_001170718 | BCAR1 | cg02328807 | 1.55913 | 0.000516496 |
| 7 |  |  | NM_207173 | NPSR1 | cg02850468 | 1.55417 | 0.00110968 |
| 22 |  |  | NM_014508 | APOBEC3C | cg11413071 | 1.54025 | 0.000294566 |
| 5 | N_Shelf | chr5:157170505-157171321 | NM_017872 | THG1L | cg22946888 | 1.52706 | 0.000403988 |
| 10 |  |  | NM_001025076 | CUGBP2 | cg05075692 | 1.5006 | 0.00532472 |
| 18 | N_Shore | chr18:32956764-32957406 | NM_145756 | ZNF396 | cg02931457 | 1.49972 | 0.001064 |
| 8 |  |  | NM_014629 | ARHGEF10 | cg15956049 | 1.48451 | 0.00238501 |
| 7 |  |  | NM_025154 | UNC84A | cg27215236 | 1.4727 | 0.00301843 |
| 7 | Island | chr7:1039874-1040096 | NM_001134395 | C7orf50 | cg21768702 | 1.46695 | 0.00254159 |
| 7 |  |  | NM_014569 | ZKSCAN5 | cg16890298 | 1.46621 | 0.000123201 |
| 5 |  |  | NM_001003841 | SLC6A19 | cg26197930 | 1.46286 | 0.00125233 |
| 7 |  |  | NM_014705 | DOCK4 | cg05046083 | 1.45984 | 0.00427552 |
| 14 |  |  | NM_031914 | SYT16 | cg14189530 | 1.445 | 0.00189709 |
| 21 |  |  | NM_015833 | ADARB1 | cg21913632 | 1.43292 | 0.00108461 |
| 10 | Island | chr10:367469-369416 | NM_014974 | DIP2C | cg01552777 | 1.43238 | 2.27E-05 |
| 17 | N_Shore | chr17:61511653-61511886 | NM_001017916 | CYB561 | cg10817916 | 1.42882 | 0.000241829 |
| 14 |  |  | NM_005197 | FOXN3 | cg23970645 | 1.40956 | 0.000849133 |
| 16 | Island | chr16:427415-427832 | NM_021259 | TMEM8A | cg02999476 | 1.40931 | 0.000334832 |
| 3 |  |  | NM_006641 | CCR9 | cg22022041 | 1.40517 | 0.000284804 |
| 12 | S_Shelf | chr12:132263617-132263856 | NM_004592 | SFRS8 | cg15832094 | 1.38896 | 0.0011102 |
| 2 | N_Shelf | chr2:45838292-45838582 | NM_018079 | SRBD1 | cg23995169 | 1.37644 | 0.000502339 |
| 7 |  |  | NM_012281 | KCND2 | cg01367751 | 1.37586 | 0.0013057 |
| 1 | Island | chr1:2537671-2537886 | NM_033467 | MMEL1 | cg19712277 | 1.36954 | 0.000113785 |
| 12 | N_Shelf | chr12:30907359-30908245 | NM_032156 | CAPRIN2 | cg18060965 | 1.36832 | 0.000199157 |
| 4 | Island | chr4:7802191-7802440 | NM_198595 | AFAP1 | cg20356136 | 1.36793 | 0.00548982 |
| 16 |  |  | NM_001080530 | SNX29 | cg00088691 | 1.36207 | 0.00189331 |
| 8 | N_Shore | chr8:15397636-15398287 | NM_178234 | TUSC3 | cg19353006 | 1.35915 | 0.000574153 |
| 10 | S_Shore | chr10:413851-414165 | NM_014974 | DIP2C | cg06920946 | 1.3589 | 0.00487198 |
| 11 |  |  | NM_002407 | SCGB2A1 | cg08459368 | 1.35747 | 0.000379738 |
| 14 |  |  | NM_014844 | TECPR2 | cg08062206 | 1.35517 | 0.0035091 |
| 7 | N_Shelf | chr7:1014698-1015239 | NM_001031617 | COX19 | cg10374962 | 1.35276 | 0.000886737 |
| 3 |  |  | NM_006810 | PDIA5 | cg27576434 | 1.35102 | 0.00297733 |
| 2 | N_Shelf | chr2:55844267-55845065 | NM_020463 | SMEK2 | cg06745378 | 1.35101 | 0.00109819 |
| 17 | S_Shelf | chr17:43238260-43239233 | NM_144608 | HEXIM2 | cg08351131 | 1.34416 | 0.0051478 |
| 11 | N_Shore | chr11:133800684-133800931 | NM_014987 | IGSF9B | cg15086884 | 1.34038 | 0.00400203 |
| 4 |  |  | NM_020894 | KIAA1530 | cg03173807 | 1.33629 | 0.00386572 |
| 6 | N_Shore | chr6:10723022-10723609 | NM_001165258 | TMEM14C | cg15936318 | 1.33436 | 0.000435471 |
| 12 |  |  | NM_017564 | STAB2 | cg03040513 | 1.33072 | 0.000464026 |
| 5 | S_Shore | chr5:169659830-169660229 | NM_001102609 | C5orf58 | cg07521912 | 1.32678 | 0.00216748 |
| 11 |  |  | NM_053005 | HCCA2 | cg24810144 | 1.32631 | 0.00163111 |
| 8 |  |  | NM_001040704 | DEFB106B | cg01683000 | 1.32489 | 0.00260979 |
| 4 | S_Shelf | chr4:148538449-148538718 | NM_018241 | TMEM184C | cg00351838 | 1.31675 | 0.00340033 |
| 17 |  |  | NM_198384 | CACNA1G | cg20271361 | 1.3164 | 0.00326893 |
| 4 |  |  | NM_005277 | GPM6A | cg08815506 | 1.3091 | 0.000107252 |
| 11 | N_Shelf | chr11:1024003-1024492 | NM_005961 | MUC6 | cg25358133 | 1.30817 | 0.00423848 |
| 7 |  |  | NM_001869 | CPA2 | cg27627209 | 1.30369 | 0.00186515 |
| 2 | N_Shelf | chr2:73495920-73496910 | NM_001080410 | FBXO41 | cg01140585 | 1.30355 | 0.00270708 |
| 7 | S_Shore | chr7:1523541-1523976 | NM_001080453 | INTS1 | cg03922095 | 1.30333 | 0.00342519 |
| 7 | S_Shelf | chr7:601121-601729 | NM_001164759 | PRKAR1B | cg08733250 | 1.30057 | 0.00296563 |
| 17 |  |  | NM_005993 | TBCD | cg21720206 | 1.29925 | 0.00241309 |
| 21 | Island | chr21:41554618-41554959 | NM_001389 | DSCAM | cg12298598 | 1.29872 | 0.000448728 |
| 13 | Island | chr13:113497296-113497661 | NM_015205 | ATP11A | cg20806040 | 1.2977 | 0.00264963 |
| 5 |  |  | NM_007118 | TRIO | cg07768372 | 1.28828 | 0.00133156 |
| 14 | N_Shelf | chr14:24550317-24551148 | NM_006032 | CPNE6 | cg12032377 | 1.28752 | 0.00341581 |
| 7 | Island | chr7:77669670-77670057 | NM_012301 | MAGI2 | cg05763154 | 1.28728 | 0.00413986 |
| 6 |  |  | NM_007028 | TRIM31 | cg04176169 | 1.28656 | 0.000836497 |
| 16 |  |  | NM_032039 | ITFG3 | cg09614814 | 1.28652 | 0.00562852 |
| 20 |  |  | NM_031229 | RBCK1 | cg08040448 | 1.28355 | 0.00246627 |
| 8 | Island | chr8:141559164-141559368 | NM_012154 | EIF2C2 | cg07242214 | 1.2816 | 0.000417841 |
| 18 | Island | chr18:580179-580899 | NM_004066 | CETN1 | cg25964180 | 1.28066 | 0.00457162 |
| 7 | N_Shore | chr7:2107080-2107381 | NM_003550 | MAD1L1 | cg01383401 | 1.2788 | 0.000857043 |
| 5 |  |  | NM_001166208 | SYNPO | cg15167202 | 1.27749 | 0.000181369 |
| 6 |  |  | NM_145176 | SLC2A12 | cg20949198 | 1.27679 | 0.00227833 |
| 7 | Island | chr7:2697934-2698204 | NM_025250 | TTYH3 | cg19339021 | 1.27547 | 0.00208678 |
| 2 |  |  | NM_006036 | PREPL | cg19752143 | 1.27509 | 0.0032676 |
| 12 |  |  | NM_002567 | PEBP1 | cg16572268 | 1.2746 | 0.00475253 |
| 1 |  |  | NM_002962 | S100A5 | cg07044523 | 1.27385 | 0.00321298 |
| 5 |  |  | NM_178276 | SERINC5 | cg07172701 | 1.27231 | 2.05E-05 |
| 13 |  |  | NM_006437 | PARP4 | cg26621704 | 1.27119 | 0.00250613 |
| 2 | S_Shelf | chr2:85197445-85199321 | NM_020122 | KCMF1 | cg13319825 | 1.27113 | 0.00250467 |
| 3 | Island | chr3:9032257-9032519 | NM_001033117 | SRGAP3 | cg17340779 | 1.27107 | 0.00177414 |
| 6 |  |  | NM_006772 | SYNGAP1 | cg20118717 | 1.27073 | 0.00102731 |
| 17 |  |  | NM_001144952 | SDK2 | cg19445578 | 1.26953 | 0.0019304 |
| 14 | Island | chr14:105836031-105836386 | NM_015197 | PACS2 | cg18853010 | 1.26701 | 0.000249646 |
| 15 |  |  | NR_026897 | MGC15885 | cg14924899 | 1.26514 | 0.00257954 |
| 7 | S_Shore | chr7:151421668-151422157 | NM_016203 | PRKAG2 | cg10902667 | 1.26312 | 0.00316305 |
| 2 |  |  | NM_018968 | SNTG2 | cg20594671 | 1.26119 | 0.000711272 |
| 16 | N_Shore | chr16:672265-672507 | NM_021168 | RAB40C | cg06219301 | 1.26043 | 0.000224665 |
| 8 |  |  | NM_004225 | MFHAS1 | cg09128169 | 1.26012 | 0.00257832 |
| 19 | N_Shore | chr19:51221447-51222704 | NM_016148 | SHANK1 | cg11801011 | 1.25907 | 0.00384145 |
| 5 | S_Shelf | chr5:36151497-36152379 | NM_032637 | SKP2 | cg24773493 | 1.25817 | 0.00313982 |
| 4 | Island | chr4:887190-888164 | NM_005255 | GAK | cg22140614 | 1.25749 | 0.00136277 |
| 18 |  |  | NM_024805 | C18orf22 | cg23745864 | 1.25665 | 0.00323908 |
| 1 | N_Shore | chr1:36038927-36040051 | NM_178548 | TFAP2E | cg00149492 | 1.25627 | 0.001871 |
| 8 |  |  | NM_004901 | ENTPD4 | cg07748154 | 1.25605 | 0.0029357 |
| 17 | Island | chr17:744617-744907 | NM_022463 | NXN | cg25717351 | 1.25481 | 0.00254489 |
| 14 |  |  | NM_001105579 | TMEM90A | cg05843944 | 1.2528 | 0.000590675 |
| 11 | N_Shore | chr11:129871690-129873423 | NM_199437 | PRDM10 | cg12409074 | 1.25148 | 0.0022999 |
| 7 | Island | chr7:1062496-1062966 | NM_001134395 | C7orf50 | cg16492851 | 1.25121 | 0.00305323 |
| 19 | Island | chr19:615691-623505 | NM_005035 | POLRMT | cg15726557 | 1.25104 | 0.0049459 |
| 17 |  |  | NM_003585 | DOC2B | cg22496001 | 1.25063 | 0.00322388 |
| 7 | Island | chr7:1062496-1062966 | NM_001134395 | C7orf50 | cg04095257 | 1.24878 | 0.00686197 |
| 15 |  |  | NM_000259 | MYO5A | cg21555346 | 1.24868 | 9.22E-05 |
| 16 |  |  | NM_013275 | ANKRD11 | cg27602981 | 1.24749 | 0.000707927 |
| 1 |  |  | NM_014849 | SV2A | cg26196882 | 1.24742 | 0.0003013 |
| 11 |  |  | NR_002569 | SCARNA9 | cg06690644 | 1.24724 | 0.00428973 |
| 7 |  |  | NM_002847 | PTPRN2 | cg25143162 | 1.247 | 0.00136255 |
| 20 | N_Shelf | chr20:33865333-33865563 | NM_006690 | MMP24 | cg07957458 | 1.2469 | 0.00126331 |
| 18 | N_Shore | chr18:70208974-70211790 | NM_182511 | CBLN2 | cg24956253 | 1.24679 | 0.00408304 |
| 4 | N_Shore | chr4:960505-960836 | NM_001347 | DGKQ | cg05178683 | 1.24632 | 0.00147521 |
| 14 |  |  | NM_007192 | SUPT16H | cg09249611 | 1.24613 | 0.00331578 |
| 10 |  |  | NM_152309 | PIK3AP1 | cg27215768 | 1.24553 | 0.00162037 |
| 17 | Island | chr17:1799541-1799756 | NM_002945 | RPA1 | cg09021817 | 1.24508 | 0.001336 |
| 1 |  |  | NM_001103 | ACTN2 | cg26406150 | 1.24387 | 0.00430807 |
| 12 | N_Shore | chr12:133303808-133304234 | NM_015114 | ANKLE2 | cg04023742 | 1.24215 | 0.000227191 |
| 7 |  |  | NM_001077664 | URGCP | cg17710536 | 1.24213 | 0.000912312 |
| 9 |  |  | NM_001077365 | POMT1 | cg14032734 | 1.24179 | 0.0024976 |
| 3 |  |  | NM_001042646 | TRAK1 | cg08228995 | 1.24178 | 0.00290631 |
| 5 |  |  | NM_013235 | RNASEN | cg15827285 | 1.24132 | 0.00293639 |
| 1 | S_Shelf | chr1:44444635-44445622 | NM_003780 | B4GALT2 | cg26272072 | 1.24131 | 0.00213205 |
| 17 | S_Shore | chr17:951746-952265 | NM_021962 | ABR | cg11933712 | 1.24112 | 0.00161549 |
| X | S_Shore | chrX:24331643-24332347 | NM_001136233 | FAM48B2 | cg12041308 | 1.23859 | 0.00312093 |
| 17 |  |  | NM_025185 | TANC2 | cg00096806 | 1.23848 | 0.001026 |
| 7 |  |  | NR_027330 | C7orf54 | cg05686445 | 1.23831 | 0.0032441 |
| 3 |  |  | NM_015199 | ANKRD28 | cg27275941 | 1.23809 | 0.00343227 |
| 2 |  |  | NR_026597 | DIRC3 | cg12596243 | 1.23567 | 0.00456386 |
| 7 | S_Shore | chr7:1970627-1970924 | NM_003550 | MAD1L1 | cg10532980 | 1.23557 | 0.000658689 |
| 21 |  |  | NM_001136130 | APP | cg21000999 | 1.23548 | 0.00243871 |
| 3 | N_Shelf | chr3:9993730-9994136 | NM_207351 | PRRT3 | cg23328707 | 1.23374 | 0.00297476 |
| 17 | S_Shore | chr17:4642038-4643410 | NM_001136046 | ZMYND15 | cg05842890 | 1.23333 | 0.00122809 |
| 10 |  |  | NM_001380 | DOCK1 | cg17335001 | 1.23238 | 0.00249917 |
| 8 | N_Shore | chr8:22547486-22553427 | NM_004430 | EGR3 | cg00732775 | 1.22994 | 0.00138866 |
| 6 | S_Shelf | chr6:32163292-32164383 | NM_004557 | NOTCH4 | cg26457116 | 1.2293 | 0.00112653 |
| 17 |  |  | NM_013276 | SHPK | cg22173019 | 1.22728 | 0.000556014 |
| 6 |  |  | NM_006913 | RNF5 | cg26981651 | 1.22455 | 0.00239182 |
| 22 |  |  | NM_004914 | RAB36 | cg17609330 | 1.22422 | 0.00142566 |
| 2 |  |  | NM_001135212 | FKBP7 | cg21749200 | 1.22404 | 0.000714062 |
| 12 | S_Shelf | chr12:53845668-53846606 | NM_001128913 | PCBP2 | cg12811072 | 1.22359 | 0.00131618 |
| 15 |  |  | NM_001114735 | BCL2A1 | cg21234506 | 1.22289 | 5.15E-05 |
| 6 | N_Shelf | chr6:3140555-3140785 | NR_026648 | BPHL | cg11587950 | 1.22186 | 0.00118318 |
| 10 |  |  | NM_021738 | SVIL | cg11794384 | 1.22078 | 0.000673491 |
| 4 |  |  | NM_198229 | RGS12 | cg16416165 | 1.22029 | 0.00259276 |
| 11 | Island | chr11:64135815-64136381 | NR_031602 | MIR1237 | cg22831978 | 1.21937 | 0.00557543 |
| 8 | N_Shore | chr8:1821602-1821914 | NM_014629 | ARHGEF10 | cg24706251 | 1.21715 | 0.00152547 |
| 8 |  |  | NM_004745 | DLGAP2 | cg26128378 | 1.21573 | 0.00330033 |
| 14 |  |  | NM_001135047 | JDP2 | cg19543317 | 1.2154 | 0.00391976 |
| 6 |  |  | NM_080679 | COL11A2 | cg05738403 | 1.21493 | 0.00397305 |
| 5 | N_Shelf | chr5:112256980-112258132 | NM_005669 | REEP5 | cg07903425 | 1.21481 | 0.00339586 |
| 7 |  |  | NM_005010 | NRCAM | cg16073236 | 1.21431 | 0.00125161 |
| 13 | N_Shelf | chr13:114165218-114165462 | NM_017905 | TMCO3 | cg12437013 | 1.21303 | 0.00192596 |
| 9 | N_Shore | chr9:137331316-137331646 | NM_002957 | RXRA | cg24164254 | 1.21262 | 0.000687446 |
| 1 | N_Shore | chr1:11589838-11590044 | NM_020780 | PTCHD2 | cg00857963 | 1.21231 | 0.00249135 |
| 12 |  |  | NM_001759 | CCND2 | cg12594237 | 1.21217 | 0.000867933 |
| 22 | S_Shore | chr22:38035350-38035928 | NM_018957 | SH3BP1 | cg13474750 | 1.21216 | 0.00210475 |
| 10 | N_Shore | chr10:134755663-134756852 | NM_173572 | C10orf93 | cg24411972 | 1.21125 | 0.00178236 |
| 2 |  |  | NM_000189 | HK2 | cg16626875 | 1.21122 | 0.00225168 |
| 16 | Island | chr16:84270421-84270857 | NM_172347 | KCNG4 | cg08070713 | 1.21107 | 0.00428061 |
| 1 |  |  | NM_001126240 | TP73 | cg14666108 | 1.21064 | 0.00415249 |
| 22 |  |  | NM_012399 | PITPNB | cg08235822 | 1.20917 | 0.00027793 |
| 12 |  |  | NM_014653 | WSCD2 | cg10955208 | 1.20903 | 0.00072896 |
| 17 |  |  | NM_000088 | COL1A1 | cg03743861 | 1.20762 | 0.000265293 |
| 7 | N_Shore | chr7:6204132-6204522 | NM_004227 | CYTH3 | cg04893402 | 1.20697 | 0.00350199 |
| 5 | S_Shore | chr5:113696516-113699195 | NM_021614 | KCNN2 | cg11712506 | 1.20614 | 0.00229906 |
| 11 |  |  | NM_004171 | SLC1A2 | cg26635576 | 1.20561 | 0.00354477 |
| 4 | Island | chr4:529848-530049 | NM_017733 | PIGG | cg04719816 | 1.20424 | 0.000392938 |
| 17 |  |  | NM_198839 | ACACA | cg01156990 | 1.20397 | 0.00353309 |
| 5 |  |  | NM_016338 | IPO11 | cg07144032 | 1.20369 | 0.00203536 |
| 1 | S_Shelf | chr1:2222198-2222569 | NM_003036 | SKI | cg16575461 | 1.20294 | 0.00222845 |
| 9 | N_Shore | chr9:130454498-130454708 | NM_001032221 | STXBP1 | cg00552574 | 1.20131 | 0.00457204 |
| 19 |  |  | NM_001161425 | ZNF610 | cg11964338 | 1.20104 | 0.000173597 |
| 2 | N_Shelf | chr2:54856098-54856487 | NM_178313 | SPTBN1 | cg05663173 | 1.20088 | 0.0017087 |
| 19 | S_Shelf | chr19:13346962-13347346 | NM_001127222 | CACNA1A | cg07855933 | 1.20065 | 0.00122652 |
| 19 | N_Shore | chr19:2235681-2237102 | NR_031596 | MIR1227 | cg24866700 | 1.2005 | 0.00224725 |
| 6 |  |  | NM_013937 | OR11A1 | cg07369956 | 1.20001 | 0.00119749 |
| *Genes with decreased methylation in HIV+/METH users* | | |  |  |  |  |  |
| 5 | N_Shore | chr5:164710-166658 | NM_052909 | PLEKHG4B | cg18816122 | -14.1354 | 0.0031854 |
| 20 | N_Shore | chr20:23015716-23016879 | NM_001052 | SSTR4 | cg01471923 | -8.32682 | 0.00206443 |
| 1 | S_Shelf | chr1:92414718-92414986 | NM_207189 | BRDT | cg01081438 | -6.46761 | 0.0035173 |
| 19 | S_Shore | chr19:23941240-23941538 | NM_138286 | ZNF681 | cg25958450 | -4.92635 | 0.00397292 |
| 2 |  |  | NM_005633 | SOS1 | cg02502145 | -4.76152 | 0.000377817 |
| 9 | S_Shore | chr9:72130887-72131100 | NM_001163 | APBA1 | cg14460215 | -4.2081 | 0.00228912 |
| 2 |  |  | NM_024027 | COLEC11 | cg10724632 | -4.07046 | 0.000147271 |
| 3 |  |  | NM_002217 | ITIH3 | cg05393861 | -3.89911 | 0.000849689 |
| 11 | Island | chr11:118938193-118938815 | NM_021729 | VPS11 | cg01385018 | -3.28687 | 0.00161723 |
| 7 |  |  | NM_020728 | ESYT2 | cg13211008 | -3.21928 | 0.00102496 |
| 2 |  |  | NM_001165963 | SCN1A | cg00881894 | -3.12914 | 0.000482399 |
| 7 | N_Shelf | chr7:16460778-16461265 | NM_001101426 | ISPD;ISPD | cg11973981 | -2.91381 | 0.00332866 |
| 6 |  |  | NM_001009991 | SYTL3 | cg06426293 | -2.90549 | 0.00545008 |
| 4 | S_Shelf | chr4:186544754-186545503 | NM_001145670 | SORBS2 | cg09120722 | -2.87212 | 0.00307104 |
| 13 |  |  | NM_000705 | ATP4B | cg06955954 | -2.62627 | 0.000246949 |
| 14 | S_Shelf | chr14:100615540-100615946 | NM_206918 | DEGS2 | cg20904336 | -2.51737 | 0.00158574 |
| 20 | Island | chr20:39316550-39319987 | NM_005461 | MAFB | cg12499119 | -2.3712 | 0.000181554 |
| 20 |  |  | NM_001098796 | TOX2 | cg26365090 | -2.3073 | 0.00065154 |
| 6 |  |  | NR_001298 | HLA-DRB6 | cg25140213 | -2.1902 | 0.00170006 |
| 6 |  |  | NM_020133 | AGPAT4 | cg09655876 | -2.16221 | 0.00151731 |
| 7 |  |  | NM_001164759 | PRKAR1B | cg10117599 | -2.16063 | 2.47E-05 |
| 20 | N_Shore | chr20:57426729-57427047 | NM_080425 | GNAS | cg23484981 | -2.15521 | 0.000348082 |
| 1 | Island | chr1:84464223-84465232 | NM_024686 | TTLL7 | cg10977910 | -2.15389 | 0.000854319 |
| 7 |  |  | NR_029411 | LOC100133091 | cg17404449 | -2.14979 | 0.000462119 |
| 15 | S_Shelf | chr15:30918312-30918565 | NM_001039841 | ARHGAP11B | cg18739374 | -2.04941 | 0.00113482 |
| 10 |  |  | NM_001143974 | ASAH2 | cg22645355 | -2.00533 | 0.00084601 |
| 6 | Island | chr6:151646668-151646958 | NM_144497 | AKAP12 | cg25855249 | -1.97173 | 0.00227179 |
| 4 | Island | chr4:76911843-76912252 | NM_018115 | SDAD1 | cg10498390 | -1.88629 | 0.0011793 |
| 21 | S_Shore | chr21:30391264-30391758 | NM_016940 | RWDD2B | cg18001427 | -1.84724 | 0.00507948 |
| 13 | N_Shore | chr13:45011151-45011395 | NM_006022 | TSC22D1 | cg23545671 | -1.80279 | 0.00223978 |
| 19 | S_Shore | chr19:48896890-48897199 | NM_000836 | GRIN2D | cg17106653 | -1.78453 | 0.00108901 |
| 19 | S_Shore | chr19:2307566-2308092 | NM_001101391 | LINGO3 | cg18796458 | -1.70527 | 0.00138856 |
| 6 |  |  | NM_001188 | BAK1 | cg01143145 | -1.65883 | 0.00207291 |
| 18 |  |  | NM_015865 | SLC14A1 | cg01016092 | -1.63774 | 1.44E-05 |
| 8 | Island | chr8:143858279-143859411 | NM_177457 | LYNX1 | cg04688051 | -1.62202 | 0.00627133 |
| 1 | Island | chr1:118727816-118728097 | NM_206996 | SPAG17 | cg01105494 | -1.60277 | 0.00219428 |
| 22 | Island | chr22:19929096-19929468 | NM_006440 | COMT | cg11032634 | -1.59644 | 0.00089186 |
| 19 | Island | chr19:8807813-8808794 | NM_178525 | ACTL9 | cg02825211 | -1.58437 | 0.000299266 |
| 22 | N_Shore | chr22:42486349-42487246 | NM_002490 | NDUFA6 | cg01529207 | -1.57994 | 0.000167915 |
| 10 | S_Shore | chr10:135048797-135052077 | NM_014468 | VENTX | cg02201753 | -1.53803 | 0.000441367 |
| 19 | Island | chr19:34972054-34973645 | NM_001080436 | WTIP | cg10771931 | -1.5343 | 0.000393398 |
| 5 | Island | chr5:140026826-140027504 | NM_002488 | NDUFA2 | cg05148093 | -1.5283 | 0.000542471 |
| 20 | Island | chr20:30192854-30194025 | NM_002165 | ID1 | cg09923107 | -1.51016 | 0.00471863 |
| 17 |  |  | NM_006987 | RPH3AL | cg04172345 | -1.4537 | 7.25E-05 |
| 11 | Island | chr11:33060699-33061846 | NM_001145541 | TCP11L1 | cg01470704 | -1.45302 | 0.00172535 |
| 13 |  |  | NM_016248 | AKAP11 | cg01952012 | -1.4429 | 0.000177098 |
| 12 | Island | chr12:96336245-96337411 | NM_182496 | CCDC38 | cg03834261 | -1.427 | 0.000149355 |
| 4 | S_Shore | chr4:185569961-185570638 | NM_152683 | CCDC111 | cg16346810 | -1.40724 | 0.00187565 |
| 1 | Island | chr1:151483573-151483902 | NM_020770 | CGN | cg15600987 | -1.40483 | 0.00248219 |
| X | Island | chrX:3263353-3265347 | NM_015419 | MXRA5 | cg08414231 | -1.39806 | 0.00169236 |
| 11 |  |  | NM_020896 | OSBPL5 | cg06120313 | -1.39053 | 0.000216255 |
| 14 | Island | chr14:90863043-90864903 | NM_001166106 | CALM1;CALM1;CALM1 | cg07150772 | -1.38557 | 0.00106889 |
| 11 | N_Shore | chr11:67764918-67765256 | NM_030930 | UNC93B1 | cg17066594 | -1.37968 | 0.00370818 |
| 15 | Island | chr15:69109708-69114001 | NM_006305 | ANP32A | cg26735215 | -1.37669 | 0.00250661 |
| 18 | Island | chr18:57363714-57365001 | NM_133459 | CCBE1 | cg16443424 | -1.37496 | 0.00303393 |
| 6 |  |  | NR_001435 | HLA-DPB2 | cg00047553 | -1.37431 | 0.000360151 |
| 4 | Island | chr4:2935461-2936974 | NR_015453 | C4orf10 | cg16176247 | -1.3726 | 0.00319528 |
| 4 | Island | chr4:166794545-166795341 | NM_012464 | TLL1 | cg12836011 | -1.37259 | 0.00388494 |
| 7 | Island | chr7:103968783-103969959 | NM_199000 | LHFPL3 | cg06493334 | -1.36428 | 0.00303693 |
| 19 | Island | chr19:49199964-49200184 | NM_000511 | FUT2 | cg16867584 | -1.36124 | 0.00200253 |
| 5 | Island | chr5:157002174-157003182 | NM_033274 | ADAM19 | cg07540118 | -1.35927 | 0.000396513 |
| 4 | Island | chr4:155471133-155471682 | NM_002669 | PLRG1 | cg20893464 | -1.35817 | 0.00302106 |
| 10 | N_Shore | chr10:15761423-15762101 | NM_003638 | ITGA8 | cg00198603 | -1.35632 | 0.00208823 |
| 12 | Island | chr12:105501333-105501908 | NM_015275 | KIAA1033 | cg10518145 | -1.35283 | 0.00423973 |
| 7 | Island | chr7:100888069-100888488 | NM_016068 | FIS1 | cg02701985 | -1.35171 | 0.00119742 |
| 3 | Island | chr3:132135973-132136619 | NM_015268 | DNAJC13 | cg05711928 | -1.34874 | 0.00269617 |
| 8 | Island | chr8:104426894-104427823 | NM_030780 | SLC25A32 | cg16930980 | -1.34697 | 0.000432137 |
| 6 | Island | chr6:10747827-10748118 | NM_030969 | TMEM14B | cg21012866 | -1.34546 | 0.00373893 |
| 16 | N_Shore | chr16:55513220-55513526 | NM_004530 | MMP2 | cg04862799 | -1.34545 | 0.00172374 |
| 18 | Island | chr18:51795711-51796332 | NM_007195 | POLI | cg02447314 | -1.34252 | 0.00532177 |
| 2 | Island | chr2:131485149-131485460 | NM_207364 | GPR148 | cg11777523 | -1.34193 | 0.00370458 |
| 1 | Island | chr1:107682889-107684463 | NM_014917 | NTNG1 | cg07005523 | -1.3405 | 0.00385704 |
| 6 | N_Shelf | chr6:4135175-4136460 | NM_206836 | PECI | cg08308214 | -1.34012 | 0.000314906 |
| 1 | N_Shore | chr1:100817709-100818899 | NM_003672 | CDC14A | cg11691902 | -1.33956 | 6.73E-06 |
| 19 | Island | chr19:45909062-45910124 | NM_012099 | CD3EAP | cg07719100 | -1.33844 | 0.000926289 |
| 4 | Island | chr4:155254130-155254603 | NM_017639 | DCHS2 | cg11934170 | -1.33814 | 0.000107159 |
| 5 | Island | chr5:10441605-10442512 | NM_031916 | ROPN1L | cg00626518 | -1.3358 | 0.00418945 |
| 1 | Island | chr1:53662309-53662815 | NM_000098 | CPT2 | cg13430225 | -1.33432 | 0.00242412 |
| 11 | Island | chr11:71791385-71791960 | NM_001145308 | LRTOMT | cg01988340 | -1.3339 | 0.00597156 |
| 21 | Island | chr21:37757496-37758113 | NM_005441 | CHAF1B | cg13854874 | -1.33368 | 0.00341367 |
| 2 | Island | chr2:219575448-219576080 | NM_014640 | TTLL4 | cg24225259 | -1.33118 | 0.00172582 |
| 16 | Island | chr16:88922671-88923976 | NM_000512 | GALNS | cg07002965 | -1.33106 | 0.000746648 |
| 12 | Island | chr12:10875137-10876180 | NM_001145426 | CSDA | cg26871120 | -1.3293 | 0.00474162 |
| 19 | Island | chr19:56111498-56112063 | NM_032836 | FIZ1 | cg08250364 | -1.32454 | 0.00536939 |
| 17 | Island | chr17:16472074-16472800 | NM_020653 | ZNF287 | cg06681220 | -1.32448 | 0.000504232 |
| 16 | N_Shore | chr16:84538884-84539115 | NM_020947 | KIAA1609 | cg09219343 | -1.32327 | 0.00106865 |
| 19 | Island | chr19:913135-913715 | NM_138774 | C19orf22 | cg01997529 | -1.32151 | 0.000723376 |
| 21 | Island | chr21:38444860-38446497 | NM_153681 | PIGP | cg24693520 | -1.32032 | 0.00352971 |
| 17 | Island | chr17:7475916-7477330 | NM_001416 | EIF4A1 | cg19913367 | -1.31871 | 0.00522998 |
| 19 | Island | chr19:40696811-40698193 | NM_002446 | MAP3K10 | cg00911192 | -1.31815 | 0.00659051 |
| 2 | N_Shore | chr2:172543901-172544690 | NM_001378 | DYNC1I2 | cg21756465 | -1.318 | 0.00379876 |
| 11 | Island | chr11:10879102-10880453 | NM_001143667 | ZBED5 | cg09338809 | -1.31544 | 0.00430589 |
| 19 | Island | chr19:1847689-1847893 | NM_020695 | REXO1 | cg23033430 | -1.31519 | 0.00467905 |
| 4 | Island | chr4:37455279-37455757 | NM_001104629 | C4orf19 | cg21131031 | -1.31404 | 0.00156644 |
| 18 |  |  | NM_030632 | ASXL3 | cg20034091 | -1.31295 | 0.000533017 |
| 5 | Island | chr5:67584213-67584451 | NM_181523 | PIK3R1 | cg07208333 | -1.31082 | 0.00430176 |
| 15 | Island | chr15:45694410-45695794 | NR_027635 | SPATA5L1 | cg25215329 | -1.3108 | 0.00141363 |
| 1 | Island | chr1:151735445-151736093 | NM_001134939 | OAZ3 | cg01717973 | -1.30968 | 0.00506987 |
| 2 | Island | chr2:166809970-166810826 | NM_024753 | TTC21B | cg10938836 | -1.3096 | 0.000733181 |
| 21 | Island | chr21:45553021-45554037 | NM_004649 | C21orf33 | cg00298153 | -1.30658 | 0.00182429 |
| 4 | S_Shore | chr4:170541251-170541810 | NM_001829 | CLCN3 | cg26171231 | -1.30508 | 0.0053254 |
| 2 |  |  | NM_014914 | AGAP1 | cg27659903 | -1.30402 | 0.00303734 |
| 1 | Island | chr1:179544720-179545307 | NM_014625 | NPHS2 | cg10980436 | -1.30327 | 0.000521466 |
| 18 | Island | chr18:23806283-23807093 | NM_005640 | TAF4B | cg11592613 | -1.3008 | 0.00309107 |
| 12 | S_Shore | chr12:113772633-113772934 | NM_024959 | SLC24A6 | cg27456220 | -1.29928 | 0.00215704 |
| 6 |  |  | NM_138700 | TRIM40 | cg08076115 | -1.29647 | 4.31E-05 |
| 1 | Island | chr1:149857769-149859470 | NM_003517 | HIST2H2AC | cg12397297 | -1.29566 | 0.00136589 |
| 8 | Island | chr8:41908373-41909581 | NM_001099413 | MYST3 | cg16743070 | -1.29514 | 0.00312769 |
| 1 | Island | chr1:182921819-182922644 | NM_030933 | C1orf14 | cg02710296 | -1.29401 | 0.00148449 |
| 1 | Island | chr1:37980071-37980565 | NM_022756 | MEAF6 | cg01711073 | -1.29288 | 0.00684936 |
| 18 | Island | chr18:51750712-51751191 | NM_003927 | MBD2 | cg16852837 | -1.29269 | 0.00407521 |
| 19 | Island | chr19:53635625-53636230 | NM_001164309 | ZNF415 | cg18301583 | -1.29156 | 0.0014076 |
| 1 | N_Shore | chr1:174968490-174969624 | NM_014412 | CACYBP | cg13384453 | -1.28991 | 0.00242457 |
| 12 | Island | chr12:50451197-50451943 | NM_020039 | ACCN2 | cg12351140 | -1.2877 | 0.00395607 |
| 1 | Island | chr1:2517397-2518892 | NM_152371 | C1orf93 | cg16599266 | -1.2858 | 0.00329192 |
| 2 | N_Shore | chr2:75937657-75938139 | NM_003203 | C2orf3 | cg22496973 | -1.28444 | 0.00209946 |
| 1 | Island | chr1:2517397-2518892 | NM_152371 | C1orf93 | cg15676078 | -1.28171 | 0.00207751 |
| 12 | Island | chr12:53894450-53895313 | NM_134324 | TARBP2 | cg04660357 | -1.28166 | 0.00274592 |
| 20 | Island | chr20:35491001-35492805 | NM_080627 | C20orf117 | cg01302165 | -1.2795 | 0.00205225 |
| 1 |  |  | NM_016448 | DTL | ch.1.4129839F | -1.27916 | 0.00328018 |
| 17 | Island | chr17:17183886-17184699 | NM_003653 | COPS3 | cg16301857 | -1.2784 | 0.00193057 |
| 17 | Island | chr17:8286376-8287336 | NM_000987 | RPL26 | cg08496503 | -1.27796 | 0.00362914 |
| 20 | Island | chr20:57463652-57467739 | NM_001077490 | GNAS | cg12372477 | -1.2778 | 0.00474952 |
| 12 | Island | chr12:104609397-104610172 | NM_001093771 | TXNRD1 | cg15459780 | -1.27547 | 0.000574616 |
| 19 | Island | chr19:821345-822669 | NM_024888 | LPPR3 | cg10213924 | -1.27452 | 0.00374526 |
| 2 | Island | chr2:71453588-71454318 | NM_020459 | PAIP2B | cg25681618 | -1.27441 | 0.00296775 |
| 19 | Island | chr19:531114-532896 | NM_004359 | CDC34 | cg17357984 | -1.2742 | 0.00134955 |
| 8 | Island | chr8:38853700-38855116 | NM_001024380 | TM2D2 | cg18497394 | -1.27357 | 0.00193917 |
| 18 | Island | chr18:35144907-35147628 | NM_001025088 | BRUNOL4 | cg18161374 | -1.27338 | 0.000496104 |
| 4 | Island | chr4:111119187-111120084 | NM_024090 | ELOVL6 | cg17484629 | -1.27038 | 0.00135794 |
| 13 | S_Shore | chr13:77566089-77566599 | NM_006493 | CLN5 | cg25287211 | -1.27 | 0.00357737 |
| 12 |  |  | NM_001024808 | BCL7A | cg07260273 | -1.2697 | 0.00387964 |
| X | Island | chrX:16729925-16730938 | NM_001144002 | CTPS2 | cg20473379 | -1.26877 | 0.00300763 |
| 13 | Island | chr13:103052361-103052944 | NM_175929 | FGF14 | cg14214706 | -1.26852 | 0.000980846 |
| 17 | N_Shore | chr17:33416129-33416578 | NM_057178 | RFFL | cg24520589 | -1.26836 | 0.00370638 |
| 3 | Island | chr3:12328994-12329227 | NM_138711 | PPARG | cg06573644 | -1.26765 | 0.00229717 |
| 7 | Island | chr7:107220344-107221075 | NR_027830 | BCAP29 | cg07196124 | -1.26698 | 0.000865788 |
| 8 | Island | chr8:26434372-26436785 | NM_001386 | DPYSL2 | cg12024050 | -1.26654 | 0.00146815 |
| 1 | Island | chr1:64058937-64059913 | NM_002633 | PGM1 | cg26108121 | -1.26597 | 0.00185114 |
| 11 | Island | chr11:65819367-65820224 | NM_006842 | SF3B2 | cg01372058 | -1.26511 | 0.00211372 |
| 8 |  |  | NM_006294 | UQCRB | cg05464172 | -1.26308 | 0.00131418 |
| 17 | S_Shore | chr17:30669000-30669287 | NM_022344 | C17orf75 | cg26741654 | -1.26283 | 0.00293815 |
| 18 | Island | chr18:31802358-31803792 | NM_003787 | NOL4 | cg20914464 | -1.26108 | 0.00318977 |
| 17 | Island | chr17:73851260-73852222 | NM_012478 | WBP2 | cg08125733 | -1.26096 | 0.00254099 |
| 20 | Island | chr20:271055-271437 | NM_153269 | C20orf96 | cg25031040 | -1.26074 | 0.000367437 |
| 14 |  |  | NM_001161726 | PPP2R5C | cg14512008 | -1.25958 | 0.00459721 |
| 2 | S_Shore | chr2:142887724-142888553 | NM_018557 | LRP1B | cg21484213 | -1.25871 | 0.00438408 |
| 19 | Island | chr19:14682657-14682994 | NM_004146 | NDUFB7 | cg03139475 | -1.25824 | 7.24E-05 |
| 7 | Island | chr7:86974740-86975088 | NR_015381 | TP53TG1 | cg19642189 | -1.25824 | 0.000922181 |
| 12 | S_Shore | chr12:31226343-31227186 | NM_004399 | DDX11 | cg27211345 | -1.25705 | 0.00480822 |
| 5 | S_Shore | chr5:141392360-141392587 | NM_005471 | GNPDA1 | cg07279858 | -1.25697 | 0.00181282 |
| 3 | Island | chr3:137906035-137906861 | NM_213654 | ARMC8 | cg05308617 | -1.25534 | 0.00121055 |
| 2 | Island | chr2:224821928-224822575 | NM_022915 | MRPL44 | cg19846927 | -1.25472 | 0.00104403 |
| 2 | Island | chr2:98962873-98964187 | NM_001298 | CNGA3 | cg02076785 | -1.25357 | 0.00131734 |
| 19 | Island | chr19:5680433-5681417 | NM_198707 | HSD11B1L | cg24348823 | -1.25152 | 0.00342191 |
| 14 | Island | chr14:50319426-50319632 | NM_004713 | SDCCAG1 | cg14513822 | -1.25137 | 0.00366544 |
| 15 | S_Shore | chr15:75660388-75661060 | NM_006715 | MAN2C1 | cg00461978 | -1.25123 | 0.00269244 |
| 10 | Island | chr10:21783198-21786420 | NM_001010911 | C10orf114 | cg10540364 | -1.24997 | 0.00265263 |
| 15 | Island | chr15:49912985-49913482 | NM_152647 | C15orf33 | cg25951582 | -1.24983 | 0.00128041 |
| 12 | Island | chr12:27863633-27864081 | NM_021821 | MRPS35 | cg10513696 | -1.24887 | 0.0044135 |
| 11 |  |  | NM_018320 | RNF121 | cg20659378 | -1.24758 | 0.00481015 |
| 10 | Island | chr10:99205476-99206274 | NM_198045 | ZDHHC16 | cg23685149 | -1.24728 | 0.00193252 |
| 17 | Island | chr17:61678154-61678678 | NM_016360 | TACO1 | cg00053292 | -1.24665 | 0.00173415 |
| 13 | Island | chr13:22177803-22178452 | NM_152726 | EFHA1 | cg24734865 | -1.24639 | 0.00396162 |
| 6 | Island | chr6:31802598-31802823 | NM_001040437 | C6orf48 | cg08566044 | -1.24633 | 2.12E-05 |
| 11 | N_Shore | chr11:47663577-47664331 | NM_014342 | MTCH2 | cg12836610 | -1.24451 | 0.00219652 |
| 2 | Island | chr2:24272582-24273313 | NM_054033 | FKBP1B | cg13401339 | -1.24311 | 0.000397022 |
| 6 | N_Shore | chr6:10887184-10887622 | NM_001040274 | SYCP2L | cg18345826 | -1.2413 | 0.004806 |
| 16 | Island | chr16:21831482-21831944 | NR_003370 | RRN3P1 | cg16426482 | -1.24086 | 0.00322304 |
| 10 | Island | chr10:35415788-35416685 | NM_183013 | CREM | cg00461005 | -1.24035 | 0.00222608 |
| 17 | Island | chr17:73401217-73401988 | NM_203506 | GRB2 | cg24771570 | -1.23898 | 0.00396957 |
| 2 | S_Shore | chr2:97405448-97405880 | NR_024521 | LMAN2L | cg01990225 | -1.23831 | 0.000236065 |
| 8 | Island | chr8:145514719-145515959 | NM_015201 | BOP1 | cg02873427 | -1.23811 | 0.00401675 |
| 6 | Island | chr6:137143635-137144445 | NM_000288 | PEX7 | cg01823693 | -1.23739 | 0.00382853 |
| 12 | N_Shore | chr12:57145948-57146333 | NM_000946 | PRIM1 | cg15809823 | -1.23548 | 0.0027338 |
| 4 | S_Shore | chr4:13485522-13486533 | NM_001159601 | RAB28 | cg23437166 | -1.23526 | 0.00301835 |
| 13 | Island | chr13:100620241-100624348 | NM_033132 | ZIC5 | cg03313945 | -1.23472 | 0.00362615 |
| 11 | Island | chr11:105481126-105481422 | NM_001077244 | GRIA4 | cg21217024 | -1.2329 | 0.00235719 |
| 2 | N_Shore | chr2:209119881-209120309 | NM_005896 | IDH1 | cg18755114 | -1.23271 | 0.000710175 |
| 1 | N_Shore | chr1:179051075-179051948 | NM_022371 | TOR3A | cg07824564 | -1.23269 | 0.00127098 |
| 12 | S_Shore | chr12:54673322-54673550 | NM_002136 | HNRNPA1 | cg25817259 | -1.23044 | 0.00131825 |
| 18 | Island | chr18:33647065-33647665 | NM_018170 | RPRD1A | cg23956068 | -1.22969 | 0.000546883 |
| 3 | N_Shore | chr3:53381345-53381936 | NM_018403 | DCP1A | cg04393905 | -1.22967 | 0.00342154 |
| 19 | S_Shore | chr19:58458686-58459219 | NM_005773 | ZNF256 | cg12048031 | -1.22898 | 0.000383972 |
| 17 | Island | chr17:38256635-38257082 | NM_021724 | NR1D1 | cg22640452 | -1.22894 | 0.00340777 |
| 5 | Island | chr5:176852953-176854125 | NM_002082 | GRK6 | cg00080123 | -1.22724 | 0.000895866 |
| 19 | S_Shore | chr19:53465730-53466323 | NM_001031665 | ZNF816A | cg02224374 | -1.22717 | 0.000982725 |
| 17 | Island | chr17:77812016-77812276 | NM_003655 | CBX4 | cg15824864 | -1.22674 | 0.00196915 |
| 13 | Island | chr13:41345062-41345570 | NM_005830 | MRPS31 | cg13368918 | -1.22513 | 0.0058045 |
| 11 | Island | chr11:47663577-47664331 | NM_014342 | MTCH2 | cg09232068 | -1.22472 | 0.00216517 |
| 3 | Island | chr3:194990987-194992478 | NM_152531 | C3orf21 | cg17057138 | -1.22376 | 0.000491195 |
| 19 | N_Shore | chr19:33571758-33572194 | NM_018025 | GPATCH1 | cg14340070 | -1.22351 | 0.00279391 |
| 19 | S_Shore | chr19:40030126-40030410 | NM_153232 | EID2 | cg22218409 | -1.2234 | 0.00153117 |
| 5 | N_Shore | chr5:11903550-11904703 | NM_001332 | CTNND2 | cg21921460 | -1.22221 | 0.00238291 |
| 14 | Island | chr14:103987063-103989796 | NM_001823 | CKB | cg14022570 | -1.22197 | 0.000660463 |
| 8 | N_Shore | chr8:53477544-53478627 | NM_207413 | FAM150A | cg05421564 | -1.22195 | 0.00176644 |
| 5 | Island | chr5:159738781-159739913 | NM_024565 | CCNJL | cg02384967 | -1.21925 | 0.00299091 |
| 14 | Island | chr14:24740564-24740942 | NM_004581 | RABGGTA | cg03056863 | -1.2189 | 0.0016006 |
| 1 | Island | chr1:32666231-32666460 | NM_024296 | CCDC28B | cg22083815 | -1.21879 | 0.00450507 |
| 11 | S_Shore | chr11:118972181-118972409 | NM_001382 | DPAGT1 | cg11506907 | -1.21877 | 0.00199279 |
| 10 | Island | chr10:11207178-11207980 | NM_006561 | CUGBP2 | cg05966978 | -1.2184 | 0.000960165 |
| X | N_Shore | chrX:70316349-70316671 | NM_005938 | FOXO4 | cg13566908 | -1.21759 | 0.00253034 |
| 12 | Island | chr12:42982832-42983881 | NM_153026 | PRICKLE1 | cg27493301 | -1.21719 | 0.00232752 |
| 7 | S_Shore | chr7:112579633-112580217 | NM_152556 | C7orf60 | cg26268016 | -1.21714 | 0.000981075 |
| 13 | Island | chr13:28542221-28543544 | NM_001265 | CDX2 | cg07311313 | -1.21703 | 0.0013606 |
| 2 | Island | chr2:217236025-217237129 | NM_020814 | "MARCH4" | cg13492070 | -1.21623 | 0.0023836 |
| 7 | S_Shore | chr7:129592032-129592891 | NM_003344 | UBE2H | cg16362141 | -1.21597 | 0.000981797 |
| 19 | Island | chr19:54494608-54494816 | NM_145815 | CACNG6 | cg03119731 | -1.21553 | 0.000604041 |
| 17 | S_Shore | chr17:7387280-7388326 | NM_020899 | ZBTB4 | cg03403113 | -1.21501 | 0.00153615 |
| 18 | N_Shore | chr18:2571286-2571580 | NM_006101 | NDC80 | cg00277996 | -1.21477 | 0.00263905 |
| 4 | S_Shore | chr4:156588297-156589292 | NM_001130683 | GUCY1A3 | cg14603466 | -1.21436 | 0.00192313 |
| 12 | Island | chr12:45609713-45610752 | NM_001142679 | ANO6 | cg07905273 | -1.21434 | 0.0005717 |
| 17 |  |  | NM_001030006 | AP2B1 | cg02186127 | -1.21303 | 0.000741335 |
| 19 | Island | chr19:59030657-59031426 | NM_032792 | ZBTB45 | cg17364234 | -1.21269 | 0.00219112 |
| 2 |  |  | NM_004882 | CIR1 | cg03350684 | -1.21258 | 0.000735308 |
| 2 | Island | chr2:20423088-20425767 | NM_001006946 | SDC1 | cg10104683 | -1.21194 | 0.000997229 |
| 1 | Island | chr1:38061428-38061740 | NM_013285 | GNL2 | cg19908139 | -1.21167 | 0.00164693 |
| 4 | Island | chr4:1005930-1006282 | NM_001004358 | FGFRL1 | cg25906220 | -1.21077 | 0.00222196 |
| 11 | N_Shore | chr11:116968849-116969512 | NM_025164 | SIK3 | cg23306832 | -1.21059 | 0.000105203 |
| 8 | N_Shore | chr8:33371897-33372526 | NM_001102401 | C8orf41 | cg04340421 | -1.20972 | 0.00174194 |
| 8 | S_Shore | chr8:41503916-41504266 | NM_152568 | NKX6-3 | cg00088007 | -1.20971 | 0.00267528 |
| 8 | Island | chr8:37654048-37656100 | NM_032777 | GPR124 | cg05613951 | -1.20915 | 0.00261876 |
| 3 | N_Shore | chr3:122102851-122103198 | NM_014367 | FAM162A | cg01689583 | -1.20854 | 0.00382689 |
| 16 | Island | chr16:30194747-30195357 | NM_007074 | CORO1A | cg08160619 | -1.20788 | 0.00460112 |
| X | Island | chrX:53253783-53254826 | NM_001146702 | KDM5C | cg12234996 | -1.20769 | 0.00216355 |
| 6 | Island | chr6:158588887-158589449 | NM_207118 | GTF2H5 | cg23612142 | -1.20766 | 0.00299565 |
| 15 | Island | chr15:45879274-45879918 | NM_012388 | PLDN | cg08624063 | -1.2076 | 0.0013229 |
| 6 | Island | chr6:143771610-143772084 | NM_003630 | PEX3 | cg11862080 | -1.20708 | 0.000357597 |
| 5 | Island | chr5:162886779-162887242 | NM_001142556 | HMMR | cg20554926 | -1.20704 | 0.00206261 |
| 4 | Island | chr4:6576735-6577173 | NM_015274 | MAN2B2 | cg03333267 | -1.20607 | 0.000584751 |
| 7 | Island | chr7:128577818-128578756 | NM_001098630 | IRF5 | cg20540942 | -1.20594 | 0.00150361 |
| 1 |  |  | NM_025207 | FLAD1 | cg27305525 | -1.20573 | 0.00206764 |
| 20 | N_Shelf | chr20:20344400-20350605 | NM_015585 | C20orf26 | cg14797413 | -1.20494 | 0.000338679 |
| 12 | N_Shore | chr12:111180006-111181028 | NM_002710 | PPP1CC | cg01613105 | -1.20384 | 0.000359875 |
| 10 | Island | chr10:104178528-104179548 | NM_002779 | PSD | cg13548361 | -1.20382 | 0.00252761 |
| 9 | Island | chr9:131314228-131315496 | NM_003127 | SPTAN1 | cg14328506 | -1.20357 | 0.003246 |
| 8 | S_Shore | chr8:29952635-29953584 | NM_015344 | LEPROTL1 | cg06927280 | -1.20264 | 0.00150389 |
| 5 | N_Shore | chr5:145718289-145720095 | NM_002700 | POU4F3 | cg14099595 | -1.20214 | 0.000249519 |
| 19 | Island | chr19:48896890-48897199 | NM_000836 | GRIN2D | cg13000134 | -1.20209 | 0.000407814 |
| 7 | Island | chr7:108209784-108210906 | NM_001130475 | THAP5 | cg09268861 | -1.20033 | 0.00442122 |
| 13 | N_Shore | chr13:37573273-37573612 | NM_001142364 | ALG5 | cg05178518 | -1.2001 | 0.00107138 |
|  |  |  |  |  |  |  |  |
